# Autoantibody-Targeted Treatments for Acute Exacerbations of Idiopathic Pulmonary Fibrosis

**DOI:** 10.1371/journal.pone.0127771

**Published:** 2015-06-17

**Authors:** Michael Donahoe, Vincent G. Valentine, Nydia Chien, Kevin F. Gibson, Jay S. Raval, Melissa Saul, Jianmin Xue, Yingze Zhang, Steven R. Duncan

**Affiliations:** 1 Department of Medicine, University of Pittsburgh, Pittsburgh, Pennsylvania, 15213, United States of America; 2 Department of Medicine, University of Texas Medical Branch, Galveston, Texas, 77555, United States of America; 3 Department of Pathology and Laboratory Medicine, University of North Carolina, Chapel Hill, North Carolina, 27599, United States of America; 4 Department of Biomedical Informatics, University of Pittsburgh, Pittsburgh, Pennsylvania, 15213, United States of America; Imperial College, London, UNITED KINGDOM

## Abstract

**Background:**

Severe acute exacerbations (AE) of idiopathic pulmonary fibrosis (IPF) are medically untreatable and often fatal within days. Recent evidence suggests autoantibodies may be involved in IPF progression. Autoantibody-mediated lung diseases are typically refractory to glucocorticoids and nonspecific medications, but frequently respond to focused autoantibody reduction treatments. We conducted a pilot trial to test the hypothesis that autoantibody-targeted therapies may also benefit AE-IPF patients.

**Methods:**

Eleven (11) critically-ill AE-IPF patients with no evidence of conventional autoimmune diseases were treated with therapeutic plasma exchanges (TPE) and rituximab, supplemented in later cases with intravenous immunoglobulin (IVIG). Plasma anti-epithelial (HEp-2) autoantibodies and matrix metalloproteinase-7 (MMP7) were evaluated by indirect immunofluorescence and ELISA, respectively. Outcomes among the trial subjects were compared to those of 20 historical control AE-IPF patients treated with conventional glucocorticoid therapy prior to this experimental trial.

**Results:**

Nine (9) trial subjects (82%) had improvements of pulmonary gas exchange after treatment, compared to one (5%) historical control. Two of the three trial subjects who relapsed after only five TPE responded again with additional TPE. The three latest subjects who responded to an augmented regimen of nine TPE plus rituximab plus IVIG have had sustained responses without relapses after 96-to-237 days. Anti-HEp-2 autoantibodies were present in trial subjects prior to therapy, and were reduced by TPE among those who responded to treatment. Conversely, plasma MMP7 levels were not systematically affected by therapy nor correlated with clinical responses. One-year survival of trial subjects was 46+15% vs. 0% among historical controls. No serious adverse events were attributable to the experimental medications.

**Conclusion:**

This pilot trial indicates specific treatments that reduce autoantibodies might benefit some severely-ill AE-IPF patients. These findings have potential implications regarding mechanisms of IPF progression, and justify considerations for incremental trials of autoantibody-targeted therapies in AE-IPF patients.

**Trial Registration:**

ClinicalTrials.gov NCT01266317

## Introduction

Idiopathic pulmonary fibrosis (IPF) is an almost invariably fatal disease with a median survival of ≤3 years.[1] IPF patients typically experience slowly progressive, if somewhat episodic, lung function deterioration. Nonetheless, a sizeable proportion of these patients, variously estimated as 10-to-50% or more, develop acute exacerbations (AE) that can result in respiratory failure and death within days.[2] No medical treatment has been shown to benefit AE-IPF patients.[1,2]

Although the pathogenesis of IPF is generally considered to be enigmatic,[1] B-cell abnormalities that are widely regarded as pathological and pathognomonic in recognized autoimmune syndromes such as systemic lupus erythematosus (SLE) and rheumatoid arthritis (RA) are also prevalent in IPF patients.[3–21] Focal B-cell accumulations in diseased organs are a prototypic characteristic of chronic adaptive immune responses to antigen(s).[22] These tissue lymphocytes not only produce antibodies (and autoantibodies), but also have numerous other immunopathogenic effects due to their elaborations of proinflammatory and vasoactive mediators.[23] Abnormal aggregates of B-cells are similarly common in IPF lungs, particularly in proximity to fibroproliferative lesions.[3–5,18–20] C-X-C motif chemokine 13 (CXCL13) is a key mediator of pathological B-cell trafficking to inflammatory foci.[24] Moreover, circulating levels of this mediator are often increased proportionately to the clinical activity of conventional autoimmune disorders.[24–27] The abnormal B-cell accumulations within damaged IPF lungs also appear to result from intrapulmonary production of CXCL13, and circulating levels of this chemokine are analogously increased and correlated with IPF manifestations, such as acute exacerbations and deaths.[18,20] Tissue deposits of antigen-antibody (immune) complexes and activated complement are highly injurious mediators of autoantibody productions in other immunological diseases,[28] and these abnormalities are also near ubiquitous in IPF lungs.[5,12,18] Increased proportions of B-cells are differentiated in patients with autoantibody-mediated disorders, including SLE, RA and Sjogren’s syndrome, due to repetitive interactions of the lymphocytes with autoantigens.[23,29,30] Similar findings are present in IPF patients, and the magnitude of their B-cell differentiation is correlated with the severity of their lung disease.[19] Circulating levels of B-lymphocyte stimulator factor (BLyS), a trophic factor critically necessary for B-cell survival and antibody production, are increased proportionately to disease activity in SLE, RA and other classical autoimmune syndromes.[31–33] BLyS levels are also abnormally increased in the pulmonary airspaces [21] and circulation of IPF patients,[19] and concentrations of the latter are associated with disease manifestations, including occurrences of acute exacerbations and mortality.

The production of antibodies with avidities for varied self-proteins is a common feature of immunological disorders, as well as being a defining criterion of “autoimmunity”.[34] Numerous distinct autoantibodies have been found in IPF cohorts,[7–17] and one or more of these self-reactive immunoglobulins are present in ≥80% of these patients.[8,12] Several specific autoantibodies have been shown to exert deleterious functional and cytopathic effects, and/or are highly associated with clinical manifestations and outcomes of IPF patients, including the development of acute exacerbations.[9–14,16,17]

Based on these reports,[3–21] and additional unpublished data, we hypothesized that autoantibodies may play a role in the progression of IPF. Other autoantibody-mediated lung diseases can also manifest with acute pulmonary dysfunction in the absence of extrinsic causes.[35–39] Lung histology in these acute cases is typified by the superimposed presence of diffuse alveolar damage, which is also a characteristic finding of AE-IPF.[2] The clinical presentations of these acute autoantibody lung syndromes are frequently indistinguishable from AE-IPF cases, and they are also resistant in most cases to treatment with glucocorticoids and nonspecific medications, again like IPF. Conversely, however, the conventional autoimmune lung disorders are often responsive to mechanistically-focused therapies that reduce autoantibodies.[35–39] A corollary of our hypothesis is that similar specific therapies might, thus, also have efficacy for AE-IPF. Accordingly, we conducted a pilot study of autoantibody-targeted treatments in critically-ill AE-IPF patients. The results of the trial indicate autoantibody-reductive therapies may benefit some patients with this otherwise refractory and highly lethal syndrome.

## Methods

The protocol for this trial and supporting TREND checklist are available as supporting information; see S1 TREND Checklist and S1 Protocol.

### Trial Subjects

Trial subjects were recruited by solicitation from among patients admitted to specialized, high-level respiratory care units between April 2011 and October 2013 at the University of Pittsburgh Medical Center (UPMC) (n = 10) or the University of Texas Medical Branch (UTMB) in Galveston, TX (n = 1).

All subjects fulfilled current consensus diagnostic criteria for IPF.[1] AE were defined by worsening hypoxemia and dyspnea within the preceding 30 days, characteristic new radiographic pulmonary infiltrates on chest CT scans obtained at hospital admission, and no other evident cause of respiratory dysfunction after thorough clinical assessments.[2]

These clinical assessments included comprehensive, replicate, and serial evaluations of historical features, physical exam findings, and laboratory data by health care teams that included experienced, specialized pulmonary medicine attending physicians, in addition to the trial investigators. The protocol for this study permitted the addition of specific autoantibody reduction modalities to standard clinical care. Standard routine tests included analyses of sputum for bacteria, fungi and respiratory viruses, blood and urine cultures, and urinary legionella antigen tests (and all of these were negative in the subjects). Other diagnostic tests were ordered by attending physicians, based on their assessments of the individual patients.

Respiratory function was too tenuous to safely permit invasive diagnostic procedures in all but two of the spontaneously breathing subjects (Subjects #8 and #11), in whom bronchoscopy with bronchoalveolar lavage (BAL) were performed. BAL was also performed in the three intubated and mechanically ventilated patients (Subjects #1,#5,#10). Studies conducted on the BAL returns were similar to those performed in the sputum specimens, and were similarly all negative. CT angiograms to exclude pulmonary emboli were performed in Subjects #1, #2, #6, and #8, and lower extremity Doppler venous studies to exclude in situ thromboses were obtained in Subjects #10 and #11. Preserved left ventricular function [e.g., that could not account for congestive heart failure or pulmonary edema] was found on cardiac ultrasounds performed in all subjects except #2 and #5, and corroborated by left heart catheterizations with pressure measurements in Subjects #3 and #7-#9).

Due to the novelty of these therapies, enrollment was limited to extremely-ill patients with rapidly progressive AE who were expected to die within days. None of the subjects were candidates for lung transplantations at their presentations.

Exclusion criteria included histories, clinical findings, or laboratory evidence of conventional autoimmune syndromes after careful evaluations that included an extensive panel of autoimmune serologic tests performed in accredited hospital clinical laboratories.[12] Other exclusion criteria for safety included a history of hepatitis B or C infection, prior adverse reaction to blood products or trial medications, prior exposures to human-murine chimeric antibodies, malignancy, ongoing treatment with a cellular immunosuppressant (e.g., cyclophosphamide, methotrexate, mycophenolate, azathioprine, calcineurin inhibitors, etc.) or angiotensin converting enzyme inhibitors, uncontrolled diabetes, hypertension or hypotension, uncorrectable coagulopathies or thrombocytopenia, or concurrent participation in other experimental trials.

Subjects #1–7 were treated on a trial protocol approved by the Institutional Review Board (IRB) at the University of Pittsburgh (U. Pitt). Experimental subjects (or their surrogates) provided written informed consent.

The trial was designed as a Phase I/II study to assess the feasibility and safety of this experimental approach in severely ill IPF patients with acute exacerbations. The primary trial endpoints were compilations of respiratory deteriorations (defined by deteriorating gas exchange) and hemodynamic deteriorations (defined as a need for medical intervention). The secondary endpoint was 60 day survival or survival to lung transplantation during this interval, compared to historical controls. Given the exploratory nature of this trial, statistical power analysis for sample size was speculative at best. However, a sample size of 10 was adopted to expose a minimum number of patients to potential risks of the experimental treatment while still obtaining useful information relevant to design of a subsequent larger trial.

Following the conclusion of the trial, and influenced by results observed in that first cohort, subjects #8–11 were treated under auspices of innovative medical practice (compassionate care). These patients gave oral consent after being fully informed of potential adverse effects and uncertain benefit with this regimen. There were no provisions for experimental blood sampling in these patients. The inclusion and analyses of their deidentified clinical data were sanctioned by additional protocols approved by the U. Pitt. and University of Texas Medical Branch (UTMB) IRBs.

### Historical Controls

Data were extracted from the Medical Archival Record System using a search query for patients admitted to PUH-UPMC with diagnoses of idiopathic pulmonary fibrosis (CPT 516.3) or post-inflammatory pulmonary fibrosis (CPT 515) during a two-year period prior to the implementation of the experimental trial. Their medical records were deidentified and reviewed in accordance with a protocol approved by the U. Pitt. IRB. The historical controls fulfilled the same inclusion and exclusion criteria as trial subjects.

### Experimental Treatments

Glucocorticoids were administered so as to not withhold “standard therapy” in the critically-ill AE-IPF trial patients, despite a lack of evidence these agents are efficacious.[1,2] The first seven subjects were treated with a conventional local regimen: methylprednisolone 1 gm i.v. on day one, followed by 40 mg/day i.v. (or the oral predisone equivalent) for two weeks (excepting rituximab treatment days), and then 20 mg i.v. (or prednisone equivalent) until day 28. The subsequent four subjects were treated with the initial methylprednisolone bolus on day one, but with a reduction of the subsequent daily doses to 20 mg/day of p.o. prednisone or equivalent i.v. methylprednisolone, to minimize steroid-associated side effects, for a total of 21 days.

Therapeutic plasma exchanges (TPE) of 1.5x the estimated plasma volumes were performed using albumin:saline (3:1), with 95–100% volume replacement. Prolongations of prothrombin times due to TPE [40,41] were treated by adjusting the replacement fluid to include fresh frozen plasma. Five (5) TPE were scheduled on treatment days 1,2,3,5 and 6 among the first seven (7) trial subjects. In some cases (Subjects #5–7), up to six additional TPE were later performed to treat clinical relapses. Based on interim consideration of clinical results, the number of scheduled TPE treatments was increased to nine (9) in the four subsequent subjects (#8–11), administered on days 1,2,3,5,6,9,11,13, and 15.

Rituximab 1 gm i.v. was administered to subjects after the fifth TPE (day 6), and either one week later among the first cohort of seven subjects, or after the final TPE (day 15) among the last four subjects. Premedications given prior to rituximab infusions were methylprednisolone 100 mg i.v., diphenydramine 50 mg i.v., and acetaminophen 650 mg p.o.

Intravenous immunoglobulin (IVIG) 0.5 mg/kg/day administrations were scheduled on days 16–19 in the last four subjects.

### HEp-2 Autoantibody Studies

Autoantibodies against HEp-2 cells were detected by indirect immunofluorescence assays (IFA) in subjects #1–7, using previously described reagents and methods.[12] Plasma specimens were obtained pretreatment, and on (±1) days 14, or at study termination or hospital discharge, and tested for these autoantibodies at dilutions of 1:20, 1:40, 1:80, and 1:160. Prior study had shown frequent false positive results among control normal plasmas at 1:10 dilutions, and no previously tested IPF specimens were positive at >1:160. Specimens were scored for the highest [most dilute] titer they were positive, determined as fluorescence intensity greater than healthy control plasmas.[12] Staining in all cases was nonspecific and distinct from the particular florescence patterns that are diagnostic of conventional autoimmune syndromes.[34]

### Matrix Metalloproteinase 7 (MMP7)

MMP7 concentrations in the plasma samples were determined by ELISA, as previously described.[42] MMP7 is a proteolytic enzyme involved in degradation of extracellular matrix, and a possible mediator of IPF.[42] MMP7 determinations were performed in the plasma specimens to examine treatment effects on concentrations of a representative soluble non-autoantibody mediator that has been linked to IPF.

### Ethics Statement

These protocols were approved by the University of Pittsburgh Investigational Review Board and the University of Texas Medical Branch Investigational Review Board.

### Statistics

Intergroup comparisons of continuous data were performed by Mann-Whitney. Dichotomous variables were analyzed by chi-square, with odds ratios (OR) and 95% confidence intervals (CI) established by logistic regression. Survival analyses were performed using product-limit estimation and comparisons by log rank. Hazard ratios (HR) and 95% confidence intervals (CI) were established by proportional hazard regression. Alpha values <0.05 were considered significant. Statistical analyses were performed using StatView (SAS Institute, Cary, North Carolina). Unless otherwise denoted, data are depicted as means±SE.

## Results

### Subjects

Demographic and clinical characteristics of the AE-IPF trial subjects are outlined in Table 1. They tended to be older (69±4 years) than historical controls (64±2 years) (p = 0.1). Males comprised 91% of the trial subjects vs. 75% of the historical controls (p = 0.3). The trial subjects all had substantial requirements for supplemental O_2_ at the time of their first experimental treatment (Table 2), and only one (Subject #8) was able to perform formal pulmonary function tests (e.g., spirometry and diffusing capacity determinations) prior to these treatments.

**Table 1:** Demographic Characteristics and Prior Histories of Trial Subjects.

Subject	Age	Gender	IPF Dx	Comments
1	76	M	5 yr	History of prior MI. AE onset 1 week prior to admission. Previous lung biopsy confirmed UIP.
2	75	F	2 months	AE onset 5 days prior to admission.
3	52	M	2 months	Severe coronary artery disease. AE onset 3 weeks prior to admission. Previous lung biopsy confirmed UIP.
4	81	M	3 yr	AE onset 2 weeks prior to admission.
5	83	M	1 yr	Precipitous AE over days (did not require supplemental O_2_ 1 week prior to admission).
6	74	M	6 months	AE onset 2 weeks prior to admission.
7	56	M	1 yr	Precipitous AE over days (did not require supplemental O_2_ 1 week prior to admission). Later lung explant confirmed UIP.
8	81	M	2 yr	AE onset <2 weeks earlier. Admitted for hospice placement.
9	52	M	2 yr	Precipitous AE over days (did not require supplemental O_2_ 1 week prior to admission).
10	61	M	1 yr	AE onset 4 weeks earlier and accelerating.
11	71	M	2 yr	AE onset 4 weeks earlier and accelerating. Previous lung biopsy confirmed UIP.

Age denoted in years. IPF Dx denotes when the original IPF diagnosis was established in these subjects, prior to their admission for acute exacerbations. AE = acute exacerbation; UIP = usual interstitial pneumonia.[1] Subjects were not listed for lung transplantation due to advanced age (Subjects #1,2,4–6,8,11), irreparable coronary artery disease (#3), or being too acutely ill to undergo the extensive necessary pre-transplantation evaluations (#7,#9). The AE-IPF in all subjects was rapidly progressive, with daily or near daily increases of hypoxemia and dyspnea. None had infections, congestive heart failure, or other causes of pulmonary dysfunction aside from the AE-IPF (2).

**Table 2:** Individual Treatment Outcomes.

#	Pre-Rx O_2_	Post-Rx O_2_	Comments
1	F_I_O_2_ 0.7PEEP 10	F_I_O_2_ 1.0PEEP 10	Lung function worsened during treatment. Died on day 11 after recurrent MI.
2	F_I_O_2_ 0.7PEEP 10	50% FM	Despite improvement, her care was changed to comfort measures only at family request. She was extubated successfully and treated with MS gtts.
3	15L NC	5L NC	Less dyspnea and ambulating after treatment. Respiratory function remainedimproved post-treatment. Died suddenly on day 76 (cause uncertain but likely cardiac).
4	10L NC	4L NC	Less dyspnea and ambulating after treatment. Respiratory function remained improved post-treatment for >365 days.
5	100% FM Bi-level positive pressure by mask	5L NC	Less dyspnea and ambulating after treatment. AE-IPF relapsed 3 days later and he required intubation and mechanical ventilation. Repeated TPE x 5, with clinical response and was successfully extubated to 5L NC. He insisted on eating but failed swallow study. He elected CMO status and ate, aspirated, and transferred to SNF on MS gtts. Died on day 32.
6	60% FM	Room Air	Much less dyspnea and ambulating after treatment, and discharged from the hospital. Relapse on day 27 and was treated again with 5x TPE, but did not respond. Died on day 40.
7	15L NC_oxi_	Room Air	Good response but disease recurred 2 d after completion of TPE x 5. Responded again to 6 additional TPE, and discharged to home on room air, where he remained until lung transplantation on day 98.
8	10L NC	3L NC	Doing well at home on day 237
9	15L NC_oxi_	Room Air	Discharged and returned to work. Awaiting transplant on day 137.
10	60% FM	F_I_O_2_ 0.7PEEP 10	Did not respond to TPE x 5 and rituximab x 1. Prostate cancer (Stage III) diagnosed by needle biopsy after treatment initiated. Support withdrawn on day 15.
11	15L NC_oxi_	1L	Discharged to rehabilitation facility. Now at home and doing well on day 96.

Pre and Post are relative to experimental treatment courses (Rx); O_2_ requirements denoted as that necessary to maintain resting arterial oxygen saturations at ≥93%; MI = myocardial infarction; FM = face mask; NC = nasal cannula; NC_oxi_ = nasal cannula with “oxymizer” reservoir; CMO = comfort measures only; MS gtts = morphine sulfate infusion; TPE = therapeutic plasma exchange; SNF = skilled nursing facility.

**Fig 1:**
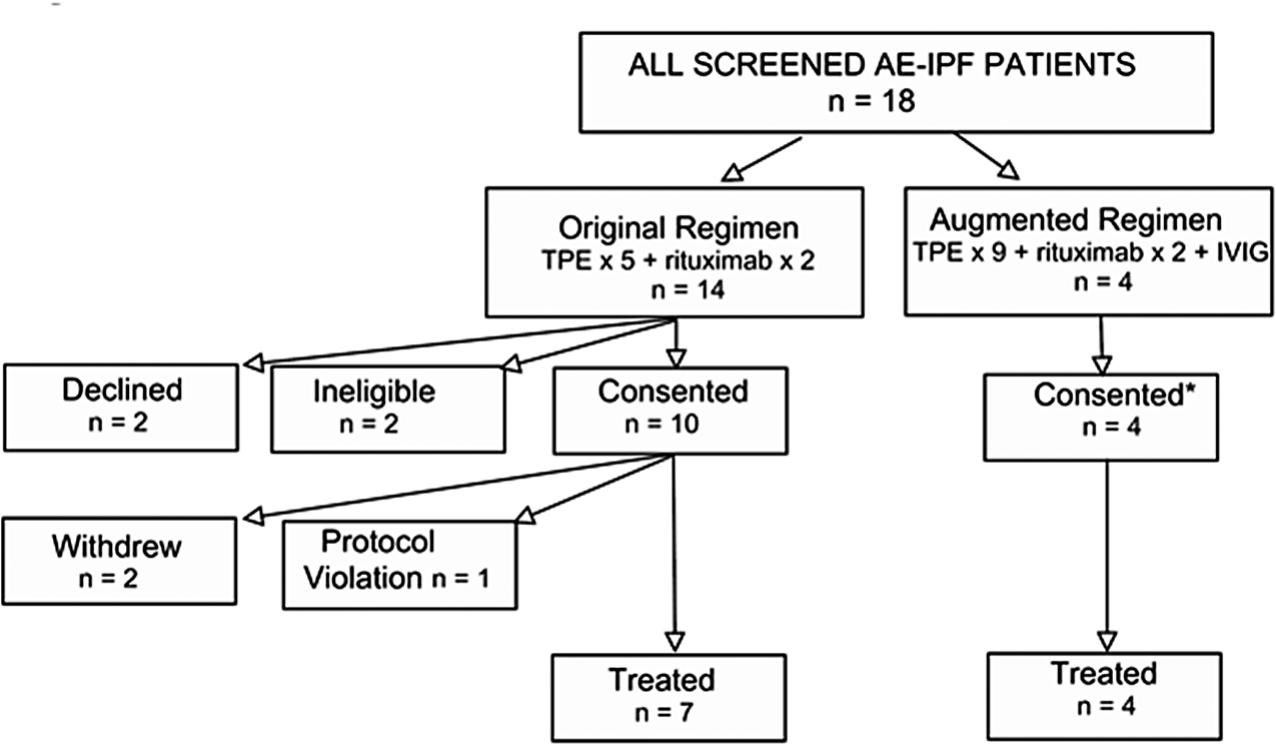
Flowchart of subject recruitments for these experimental trials. Original Regimen denotes the first series of subjects who were treated with the initial, relatively more conservative regimen (#1–7). Augmented Regimen denotes the most recent four subjects (#8–11) who received a more aggressive therapeutic course, based on interval results in the first cohort (see text for details). TPE = therapeutic plasma exchange; IVIG = intravenous immunoglobulin; * denotes oral consent of patients, under auspices of innovative clinical practice, that were given by these patients after being fully informed of potential risks and yet-unproven efficiencies of the novel treatments.

Of eighteen AE-IPF patients screened for the trial (Fig 1), two declined to participate, and two others were not eligible due to a preexistent history of cancer or an unwillingness to fully comply with treatments. Two other subjects withdrew from the study soon after giving informed consent, but before their treatments, due to very rapid deteriorations and their unwillingness to continue supportive medical care (e.g., intubation and institution of mechanical ventilation). Given that the primary focus of this study was to examine feasibility and safety of the treatment regimen, details of these subjects are not included in subsequent analyses, unless otherwise denoted. All six of these screened but untreated subjects died within 30 days.

Another subject gave informed consent, after clinical evaluation and a favorable screening review of recent medical tests performed at another institution. Repeat testing here, however, with results that became available after experimental treatment had started, showed he had positive autoantibody assays for antinuclear antigens and rheumatoid factor. This subject was judged to be a protocol violation and was excluded from further study (Fig 1).

### Clinical Observations

**Fig 2:**
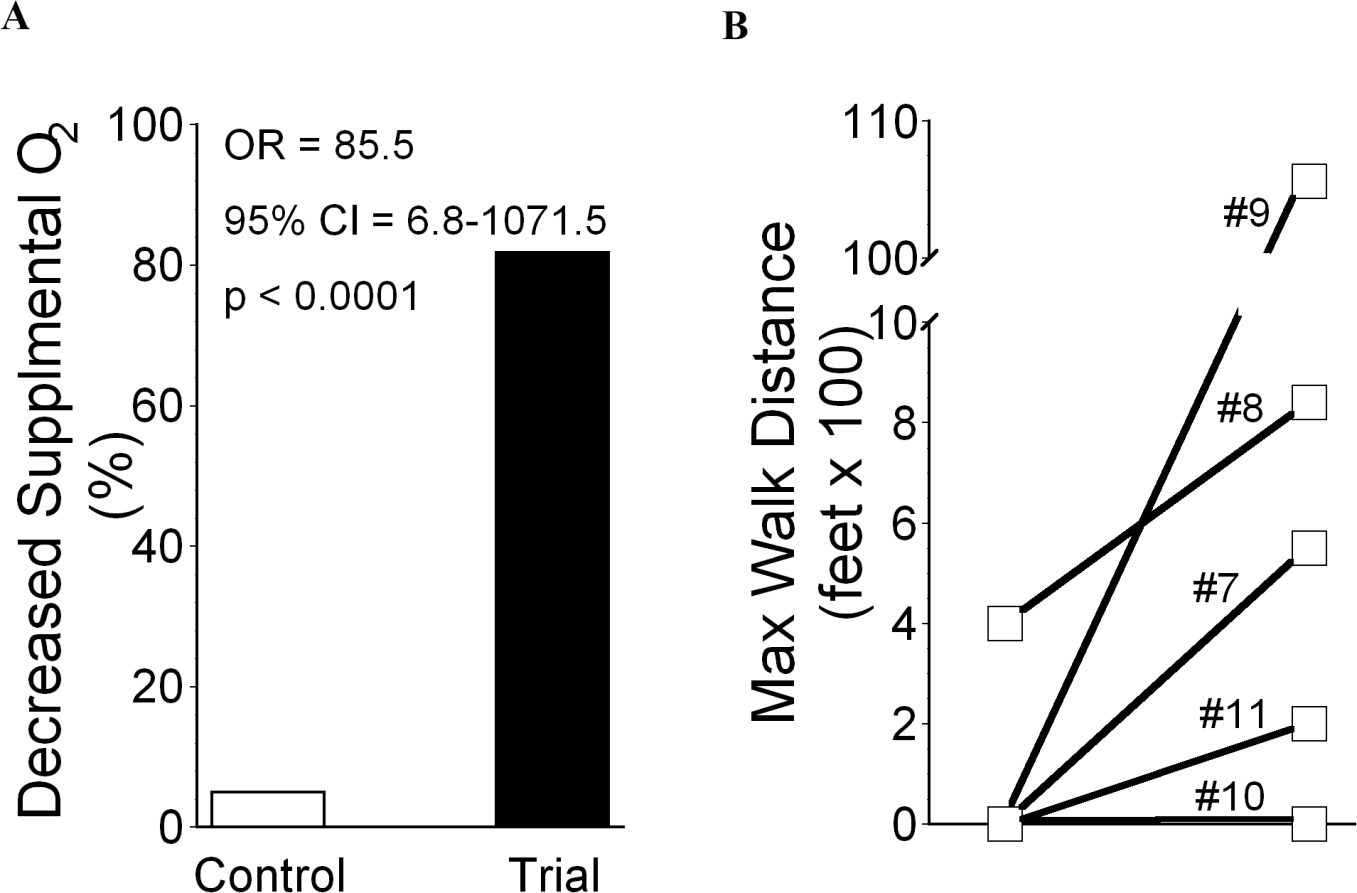
Clinical Responses to Experimental Therapy. A.) Decreases in Supplemental Oxygen Requirements. Oxygen requirements decreased in only one of the historical control subjects during their hospitalizations, whereas pulmonary gas exchange improved with experimental treatments among most of the trial cohort (see also Table 2). B.) Changes in Abilities to Ambulate. Trial subjects who responded to experimental therapy reported improved exercise tolerance, but maximal walk distances were added as a formal outcome assessment in latest subjects, identified here by subject number (see Tables 1 and 2). With the exception of Subject #10 who showed no response to the experimental treatment, the walk distances of these later patients increased substantially. (Note: The post-treatment >2 mile distance of Subject #9 was limited by boredom rather than exercise capacity).

**Fig 3:**
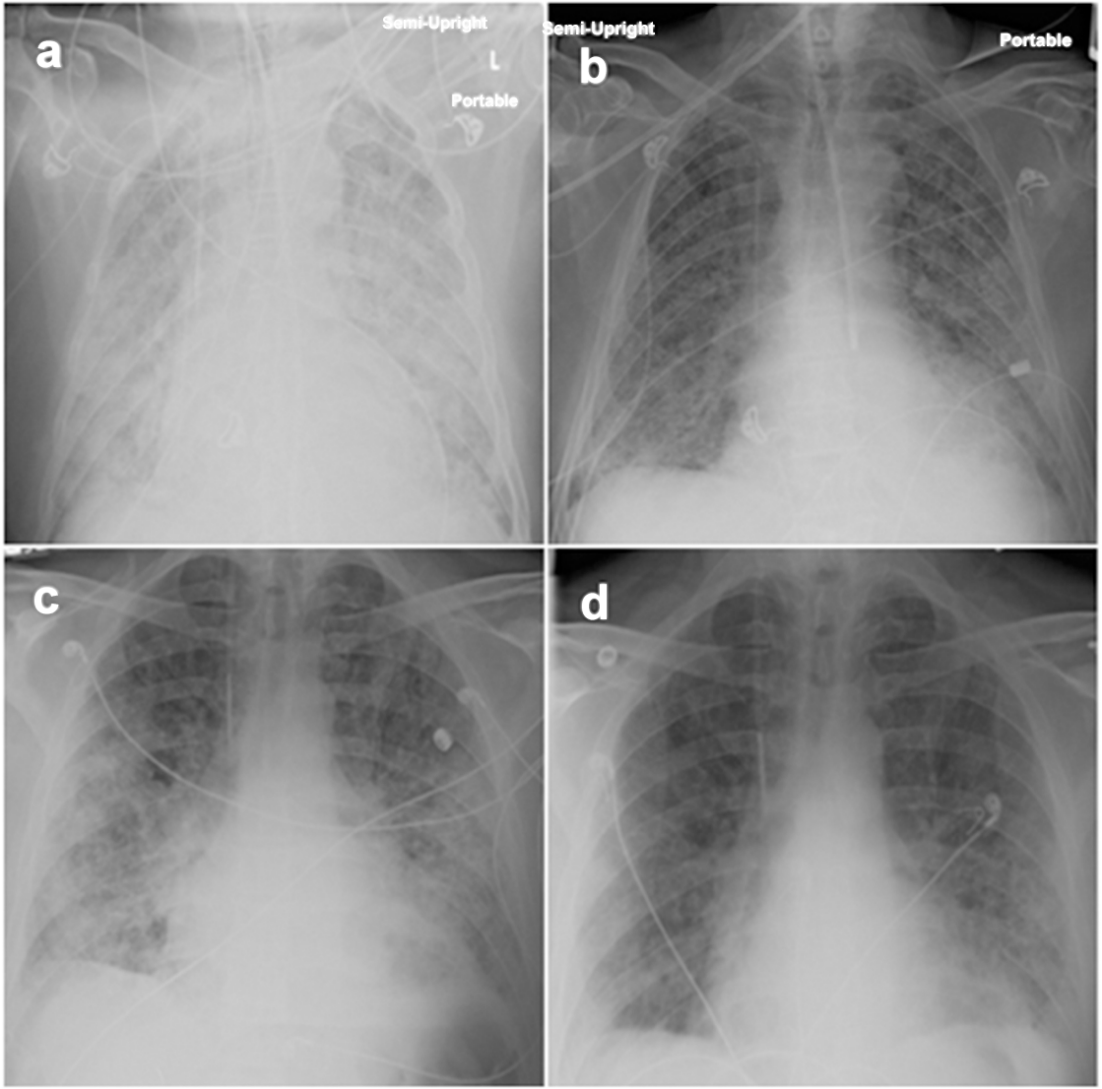
Radiographic Changes with Experimental Treatment. In addition to better lung function and gas exchange, experimental treatments frequently improved the chest radiographs (CXR) of these subjects. a) Pretreatment CXR of Subject #5 during relapse shows diffuse infiltrates; b.) Radiographic improvement (and extubation) of this subject to 2^nd^ TPE series. c.) CXR of Subject #7 immediately prior to treatment and d.) after first three TPE. These (and other) radiographic and clinical improvements in the subject population were not attributable to changes in intravascular volume status or infection.

A priori intentions to measure gas exchange as arterial oxygen partial pressures (P_a_O_2_) while subjects breathed 100% oxygen (F_I_O_2_ = 1.0) were precluded by the inabilities of many air-hungry patients to tolerate the requisite tight-fitting face masks, and dependence of an early subject on noninvasive (face mask) bi-level ventilator support (which confounds interpretations of P_a_O_2_/F_I_O_2_). Nonetheless, all but two of the experimental trial subjects (#1 and #10) had obvious improvements of gas exchange after TPE treatments (Table 2), in distinction to effects of conventional steroid therapy among historical controls (Fig 2A). Treatments among all the spontaneously breathing subjects except #10 resulted in subjective reports of less dyspnea and greater exertional tolerance. Maximal walk distances pre- and post-treatment were measured in Subjects #7–11 (Fig 2B). Total lung capacity of Subject #8 increased from 41% of predicted to 52% after therapy; respective measures of diffusing capacity also improved from 20% to 33% of predicted. Clinical responses were also accompanied by radiographic improvements (Fig 3).

Despite clinical improvement of Subject #2, manifested by decreased requirement for supplemental oxygen, successful removal from mechanical ventilation, and extubation, her medical power of attorney insisted on withdrawal from the study and institution of comfort measures only. Three subjects experienced relapses after their initial course of five TPE (#5–7). Repeat TPE again resulted in substantial improvement in two of these subjects (#5 and #7) (Table 2). There have been no relapses among the three subjects (#8,#9,#11) who responded to the augmented treatment regimen (nine TPE + rituximab + IVIG) (Table 2).

TPE was well-tolerated in spontaneously breathing patients. Short-term phenylephrine infusions for transient hypotension were necessary in two of three subjects who had TPE during mechanical ventilation (#1 and #2). Subject #6 developed intertriginous candida dermatitis, possibly related to steroid therapy, which was successfully treated with anti-mycotic agents. Increased serum glucose levels (managed with short-term insulin administrations) were frequent among the first cohort of seven subjects treated with the higher steroid dose regimen. There were no adverse events attributable to the rituximab or IVIG.

### Autoantibodies

**Fig 4:**
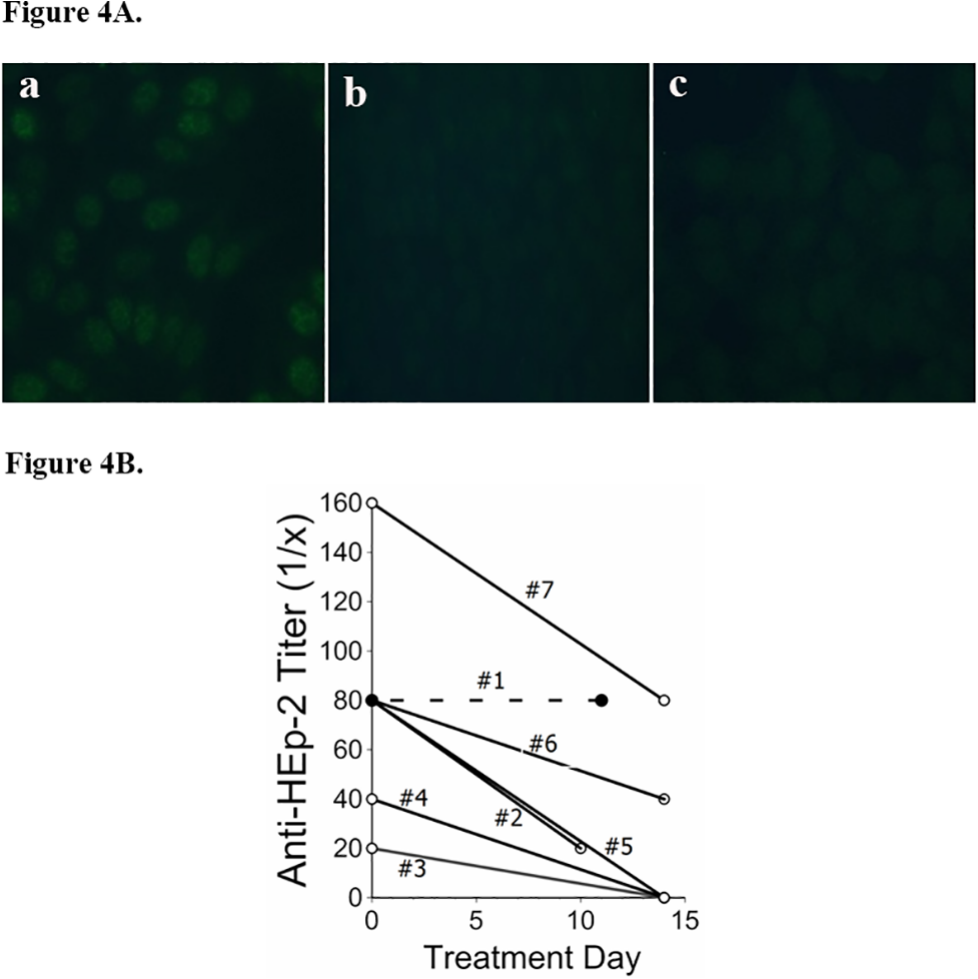
Anti-HEp-2 Autoantibodies in Trial Patients. A.) Indirect Immuno-fluorescence Assays (IFA). a.) Pretreatment anti-HEp-2 autoantibodies were present at plasma titrations of 1:80 in Subject #6. b.) Autoantibodies were diminished immediately following his TPE treatment (image here at 1:20 titration). c.) Normal plasma control specimen (1:20 titration). B.) IFA Titers. Anti-HEp-2 autoantibody titers were determined in the first eight subjects. Titers were reduced following the initial TPE series in all except Subject #1 (dotted line, solid circles), who was also the only subject among these that did not have a beneficial clinical response.

Anti-HEp-2 autoantibodies were present prior to treatment in the eight subjects in whom these immunoglobulins were assayed, and all among these except Subject #1 had reductions of Anti-HEp-2 titers following TPE (Fig 4). Subject #1 was also notable for his refractoriness to therapy. The two subjects with prolonged treatment responses after only five TPE (#3 and #4) had lower initial anti-HEp-2 titers, as well as no detectable autoantibodies after treatment.

### MMP7

**Fig 5:**
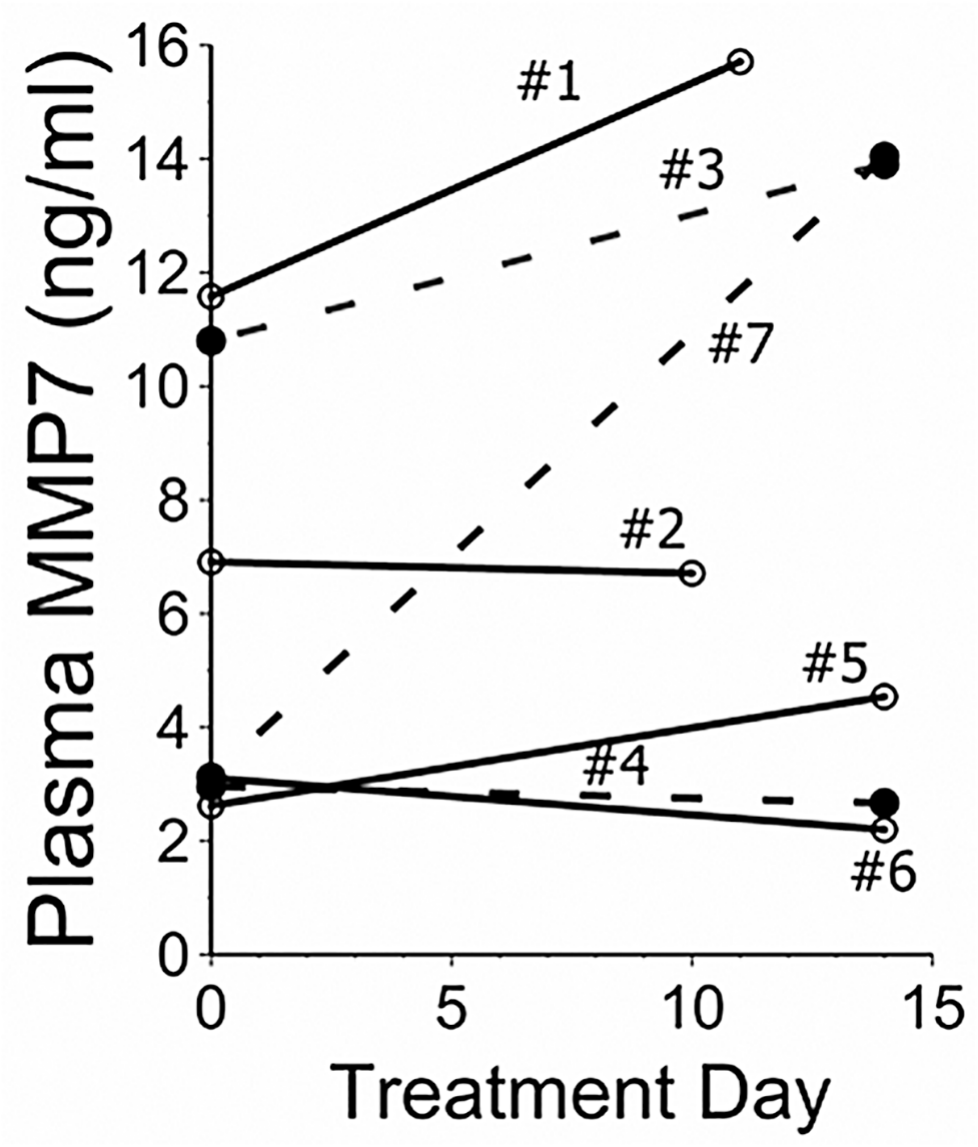
Matrix Metalloproteinase 7 (MMP7). Plasma MMP7 levels did not appear to consistently decrease with TPE, nor correlate with clinical responses. In particular, three subjects (#3,4,7, open circles, dotted lines) had increases or no changes of MMP7 levels from pretreatment values despite having prolonged clinical remissions.

MMP7 levels were not consistently altered by TPE, nor overtly correlated with clinical responses (Fig 5).

#### Survival

Sixty (60) day survival of the experimental subjects was 55 ± 15%, compared to 20 ± 9% in the AE-IPF historical control group (HR = 0.35, 95% CI = 0.13–0.98, p = 0.035). Although assessment of longer-term survival was not an a priori endpoint, the unexpectedly prolonged responses of many experimental trial subjects prompted us to compare survival over longer intervals.

**Fig 6:**
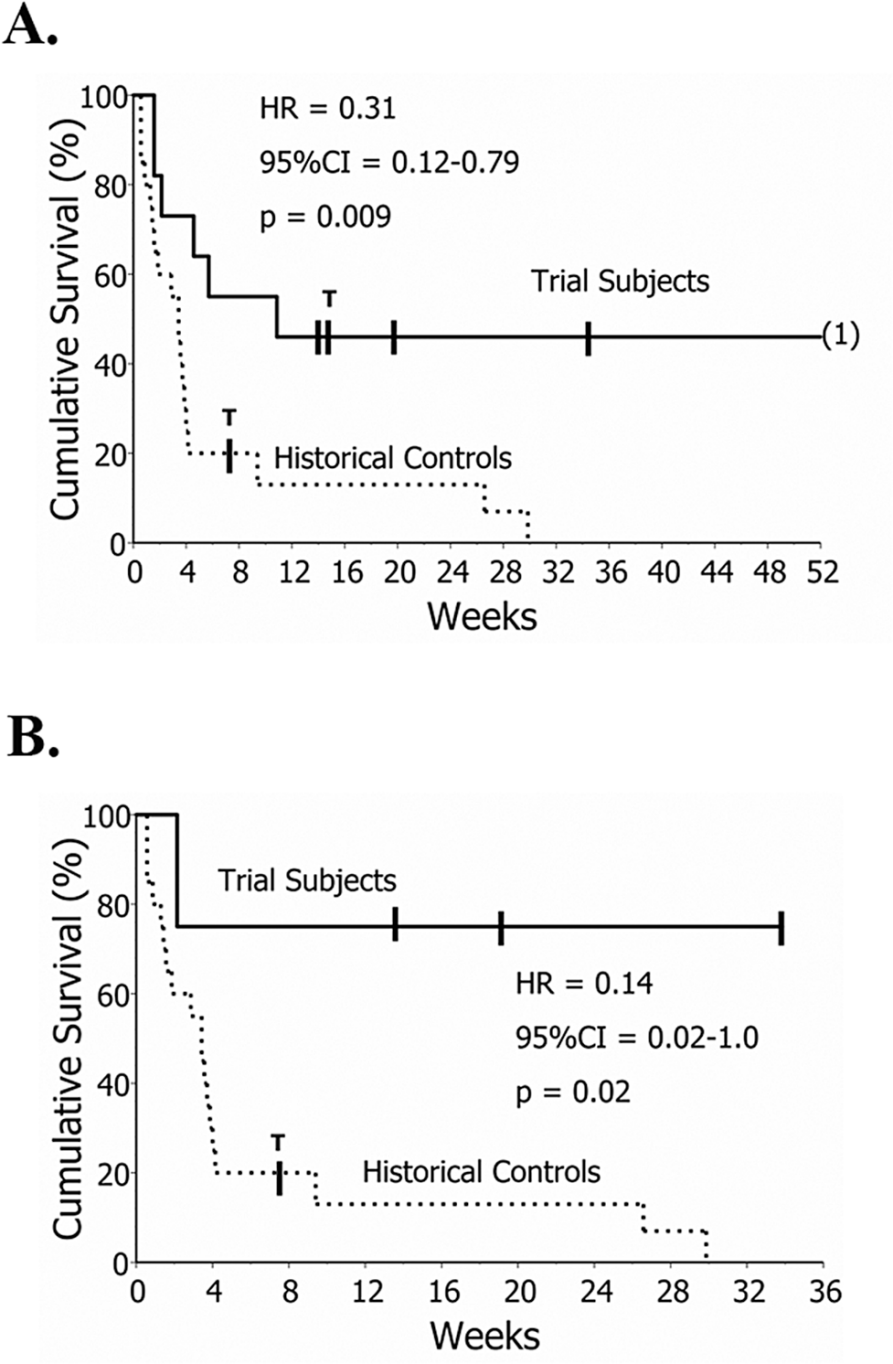
Survival Comparisons. A.) Survival in the aggregate trial subject population (n = 11) was greater than among historical control AE-IPF patients (n = 20). Cross hatches and numbers in parentheses denote censored observations. Lung transplantation censoring is denoted with “T”. B.) Clinical responses may be more durable and survival may be further enhanced among the later trial subjects (n = 4) treated with a more aggressive regimen of autoantibody-targeted modalities (9 initial TPE + rituximab + IVIG).

The aggregate experimental trial cohort had significantly better one-year survival than historical controls (Fig 6A). Results to date also suggest the later, more aggressive treatment protocol (nine TPE + rituximab + IVIG), may result in more durable responses (Fig 6B).

These analyses included the first two trial subjects who were intubated at enrollment (AE-IPF patients who require mechanical ventilation have singularly poor prognoses [2]), and the subject with an underlying malignancy (discovered after the experimental treatment was initiated), which would have been an exclusion criterion (cancer patients often develop refractory autoimmune syndromes [43]). The analyses here also included the two subjects who received a treatment course, and responded to same, but nonetheless withdrew from the trial protocol and further supportive medical care (#2 and #5) (Table 2).

Inclusion of data from all eligible IPF patients (intent-to-treat), including the two enrolled IPF subjects who did not receive a therapeutic course prior to their withdrawal (Fig 1) still shows a one-year survival advantage of these treatments (39 ± 14%) vs. the historical controls (0%) (HR = 0.42, 95%CI = 0.18–0.99, p = 0.038).

## Discussion

Given many parallels between classical autoimmune syndromes and IPF [3–21,44–46], we hypothesized specific treatments that reduce autoantibodies might also benefit AE-IPF patients. No inference can yet be drawn as to whether or not autoimmunity, defined by the presence of autoantibodies [7–17] and autoreactive T-cells,[8,12,13] is a primary cause of the IPF, or is instead a “secondary” response that develops subsequent to a distinct initial injury[43,47,48] or other dysfunctional consequence of aging (e.g., immunosenescence).[49,50] Nonetheless, the favorable effects of focused autoantibody-reduction therapies in many of the trial subjects here are at least an indication that autoantibodies might play a role in AE-IPF [3–21].

Among these therapies, TPE rapidly reduces circulating autoantibodies,[40,41,51] and was employed here to achieve swift effects in the very rapidly progressing patients. Since this was a novel therapy for AE-IPF, the initial protocol regimen of five TPE (used in Subjects #1–7) was predicated on a conservative a priori consideration that aimed to balance the potential to show some clinical effects, while minimizing the risks and expenses of a wholly untested modality. While TPE rapidly reduces autoantibodies in the circulation, however, the majority of immunoglobulins at any given moment are localized in extravascular tissue and are inaccessible to removal by plasma filtration.[41] These extravascular autoantibodies subsequently re-equilibrate down concentration gradients back into the circulation after TPE treatments, but this is rate-limited, and significant depletion of total body autoantibodies typically requires numerous treatments over several days. Thus, clinical relapses are common after therapy of other life-threatening autoantibody diseases when TPE is terminated prematurely, and effective management of these patients often requires far more than five treatments. Given the absence of benefit in Subject #1, in whom autoantibody titers were unaffected by five TPE, as well as the favorable responses to additional plasma exchanges of two relapsing subjects (#5 and #7), a greater number of TPE treatments was administered in later subjects (#8–11), with seemingly more prolonged benefit (Fig 6B).

The present data indicate clinical responses of AE-IPF patients to TPE may correlate with reductions of pre-treatment autoantibody titers (Fig 4B). In contrast, the absence of an association between experimental treatments and MMP7 concentrations, or correlations between the latter and clinical courses, indicates the patient responses are not simply attributable to TPE removal of circulating [non-autoantibody] injury mediators (Fig 5). Moreover, TPE effects on concentrations of circulating cytokines, proteases, and other mediators are known to be transient,[40,41] and cannot plausibly account for the prolonged remissions of many trial subjects.

The duration of benefit following a TPE course is ultimately limited due to the continued antibody production of autoreactive B-cell clones. Accordingly, TPE was supplemented with rituximab, a cytolytic, anti-B-cell chimeric murine-human monoclonal antibody with proven efficacy in many other autoantibody-mediated diseases,[36–39]. The onset of rituximab effects on circulating autoantibodies may be delayed for weeks after administration, however, which would seem to preclude solo therapy with this agent in rapidly progressive AE-IPF patients who would almost certainly die in the meantime.

Acute reductions of circulating immunoglobulins by TPE may also remove Fc-receptor-mediated feedback inhibition of the autoreactive B-cells that escape depletion therapies (e.g., ritixumab).[51] These lymphocytes can consequently increase their production of deleterious autoantibodies, resulting in rapid rebounds of autoantibody concentrations back to or even greater than pre-treatment levels. IVIG is widely used in the management of other serious autoimmune disorders, as an adjunct with TPE and/or rituximab, to mitigate autoantibody rebound and/or provide additional immunomodulatory effects.[51,52] IVIG but was not employed in the first series of subjects, again due to our initial bias for conservatism given the novelty of this treatment approach for AE-IPF.

Whether the more consistently prolonged response of the latest subject cohort (Fig 6B) is due to the use of a greater number of initial TPE (nine treatments) or is instead attributable to the addition of IVIG (or synergism between the two modalities) awaits additional study. The appropriate role of steroids as an adjunct to the more specific autoantibody-focused treatments similarly cannot be ascertained by the present study. Steroids have no known efficacy in AE-IPF, but for practical purposes are standard of care, given the absence of any known effective treatment for this syndrome, and despite the propensity of these agents for causing infectious and metabolic complications.[1,2]

There are several limitations of this study. This was a pilot, open-label trial of an unprecedented regimen for a highly lethal disease and, as such, included only small numbers of subjects and historical controls. Moreover, the trial regimen evolved to more aggressive therapy in later patients after observing early relapses among some of the first subject cohort. Our desire to provide what increasingly appeared to be potentially life-saving therapy for our later patients overrode concerns for conducting a “clean” experiment using an unchanging and seemingly suboptimal initial medical intervention that had been designed without prior experience, and had been biased for conservatism. This study was intended to gain incremental insights relevant to a novel approach for therapy of extremely-ill patients, for whom we also had [even greater] responsibilities as medical care providers. It was not designed to result in the definitive evidence of a Phase III trial.

Despite these short-comings, the often striking and unprecedented results of the pilot trial are an encouraging indication that this highly morbid and heretofore untreatable syndrome may be amenable, at least in some cases, to specific autoantibody-targeted therapies. Although there is little reason to believe these modalities will reverse the underlying chronic lung fibrosis of the subjects, the development of therapies that reduce discomfort and extend lives of terminally-ill patients is still laudable, and the goal of considerable effort in other disciplines (e.g., oncology). AE-IPF patients are also often too severely-ill at their presentation to undergo de novo lung transplantation evaluations (e.g. cardiac catheterizations, colonoscopies, swallow studies, etc.), or survive long enough for a donor organ to become available. Thus, clinical improvements effected by these treatments may also be a useful “bridge to transplantation”, as they were here for Subject #7 and may yet be for Subject #9 (Table 2).

We believe these initial results justify subsequent incremental studies of autoantibody-reduction therapies in AE-IPF patients. These could include trials with concurrent, conventionally-treated randomized control subjects, in order to more conclusively prove the effectiveness of autoantibody-targeted interventions. Further studies could also refine treatment regimens, and evaluate the utility of longitudinal autoantibody measures to personalize these treatments. Given minimal advances in the management of IPF, which remains a highly morbid and almost invariably fatal disorder despite decades of intense investigation,[1,2] objective considerations of novel pathogenic paradigms and treatments seem especially warranted.

## Supporting Information

S1 Protocol Trial Protocol. (DOC)

S1 TREND Checklist TREND checklist. (PDF)
