# Correction: Autoantibody-Targeted Treatments for Acute Exacerbations of Idiopathic Pulmonary Fibrosis

**DOI:** 10.1371/journal.pone.0133684

**Published:** 2015-07-20

**Authors:** Michael Donahoe, Vincent G. Valentine, Nydia Chien, Kevin F. Gibson, Jay S. Raval, Melissa Saul, Jianmin Xue, Yingze Zhang, Steven R. Duncan

In the penultimate sentence of the Results section of the Abstract, the plus sign should be a plus-or-minus sign. The correct sentence is: One-year survival of trial subjects was 46±15% vs. 0% among historical controls.

In Table 2, spaces are missing before the acronym “PEEP” in the “Pre-Rx O_2_” and “Post-Rx O_2_” columns. Please see the correct Table 2 here.

**Table 2:** Individual Treatment Outcomes.

#	Pre-Rx O_2_	Post-Rx O_2_	Comments
1	F_I_O_2_ 0.7 PEEP 10	F_I_O_2_ 1.0 PEEP 10	Lung function worsened during treatment. Died on day 11 after recurrent MI.
2	F_I_O_2_ 0.7 PEEP 10	50% FM	Despite improvement, her care was changed to comfort measures only at family request. She was extubated successfully and treated with MS gtts.
3	15L NC	5L NC	Less dyspnea and ambulating after treatment. Respiratory function remainedimproved post-treatment. Died suddenly on day 76 (cause uncertain but likely cardiac).
4	10L NC	4L NC	Less dyspnea and ambulating after treatment. Respiratory function remained improved post-treatment for >365 days.
5	100% FM Bi-level positive pressure by mask	5L NC	Less dyspnea and ambulating after treatment. AE-IPF relapsed 3 days later and he required intubation and mechanical ventilation. Repeated TPE x 5, with clinical response and was successfully extubated to 5L NC. He insisted on eating but failed swallow study. He elected CMO status and ate, aspirated, and transferred to SNF on MS gtts. Died on day 32.
6	60% FM	Room Air	Much less dyspnea and ambulating after treatment, and discharged from the hospital. Relapse on day 27 and was treated again with 5x TPE, but did not respond. Died on day 40.
7	15L NC_oxi_	Room Air	Good response but disease recurred 2 d after completion of TPE x 5. Responded again to 6 additional TPE, and discharged to home on room air, where he remained until lung transplantation on day 98.
8	10L NC	3L NC	Doing well at home on day 237
9	15L NC_oxi_	Room Air	Discharged and returned to work. Awaiting transplant on day 137.
10	60% FM	F_I_O_2_ 0.7 PEEP 10	Did not respond to TPE x 5 and rituximab x 1. Prostate cancer (Stage III) diagnosed by needle biopsy after treatment initiated. Support withdrawn on day 15.
11	15L NC_oxi_	1L	Discharged to rehabilitation facility. Now at home and doing well on day 96.

Pre and Post are relative to experimental treatment courses (Rx); O_2_ requirements denoted as that necessary to maintain resting arterial oxygen saturations at ≥93%; MI = myocardial infarction; FM = face mask; NC = nasal cannula; NC_oxi_ = nasal cannula with “oxymizer” reservoir; CMO = comfort measures only; MS gtts = morphine sulfate infusion; TPE = therapeutic plasma exchange; SNF = skilled nursing facility.
