# Supplementary material for: Autoantibody-Targeted Treatments for Acute Exacerbations of Idiopathic Pulmonary Fibrosis
Source: PLoS One. 2015 Jun 17;10(6):e0127771. doi: 10.1371/journal.pone.0127771 (PMC4470587; doi:10.1371/journal.pone.0127771)
Supplement: S1 Protocol — (DOC) [file pone.0127771.s001.doc]

**Supportive Information File 1 (S1). Trial Protocol**

Open-Label, Feasibility Study of Combined Plasma Exchange (PEX), Rituximab, and Corticosteroids in Patients with Acute Idiopathic Pulmonary Fibrosis Exacerbations

**Michael Donahoe, MD**

Principal Investigator

**Steven Duncan, MD**

Co-Principal Investigator

**Kevin Gibson, M.D**

Co-Principal Investigator

University of Pittsburgh

School of Medicine

Division of Pulmonary, Allergy, and Critical Care Medicine

Version 1.0

November 8, 2010

**Table of Contents**

[1**.** STUDY OBJECTIVE, SPECIFIC AIMS, BACKGROUND AND SIGNIFICANCE](#__RefHeading___Toc146006015) 3

[1.1 OBJECTIVE](#__RefHeading___Toc146006016) 3

[1.2 SPECIFIC AIMS](#__RefHeading___Toc146006017) 3

[1.3 BACKGROUND](#__RefHeading___Toc146006018) 4

[1.4 SIGNIFICANCE 1](#__RefHeading___Toc146006018)5

[2. RESEARCH DESIGN AND METHODS 1](#__RefHeading___Toc146006022)5

[2.1 CLASSIFICATION AND METHODOLOGICAL DESIGN 1](#__RefHeading___Toc146006016)5

[2.2 DETAILED DESCRIPTION OF RESEARCH ACTIVITIES 1](#__RefHeading___Toc146006018)5

[2.3 CRITERIA (i.e., endpoints)](#__RefHeading___Toc146006016) 18

[2.4 STATISTICAL APPROACH](#__RefHeading___Toc146006018) 19

[3. HUMAN SUBJECTS 2](#__RefHeading___Toc146006026)0

[3.1 SUBJECT POPULATION 2](#__RefHeading___Toc146006018)0

[3.2 INCLUSION CRITERIA](#__RefHeading___Toc146006018) 21

[3.3 EXCLUSION CRITERIA](#__RefHeading___Toc146006018) 21

[4. RECRUITMENT AND INFORMED CONSENT PROCEDURES](#__RefHeading___Toc146006030) 22

[4.1 RECRUITMENE METHODS](#__RefHeading___Toc146006018) 22

[4.2 INFORMED CONSENT PROCEDURES](#__RefHeading___Toc146006018) 22

[5. POTENTIAL RISKS AND BENEFITS](#__RefHeading___Toc146006030) 23

[5.1 POTENTIAL RISKS 23](#__RefHeading___Toc146006018)

[5.2 ALTERNATIVE TREATMENTS 2](#__RefHeading___Toc146006018)5

[5.3 BENEFITS 2](#__RefHeading___Toc146006018)5

[5.4 RISK MANAGMENT 2](#__RefHeading___Toc146006018)5

[5.5 DATA SAFETY MONITORING PLAN 2](#__RefHeading___Toc146006018)9

[6. COSTS AND PAYMENTS 30](#__RefHeading___Toc146006030)

[6.1 COSTS 3](#__RefHeading___Toc146006018)0

[6.2 PAYMENTS 30](#__RefHeading___Toc146006018)

[7. QUALIFICATIONS AND SOURCE OF SUPPORT](#__RefHeading___Toc146006030) 31

[7.1 QUALIFICATIONS OF INVESTIGATORS](#__RefHeading___Toc146006018) 31

[7.2 SOURCE OF SUPPORT](#__RefHeading___Toc146006018) 31

[8. REFERENCES](#__RefHeading___Toc146006052) 32

**RESEARCH PROTOCOL ABSTRACT:**

This is an open-label Phase I/II trial to assess the feasibility and safety of combined plasma exchange (PEX), rituximab, and conventional corticosteroid administration on the outcome of hospitalized patients with acute IPF exacerbations. The specific aims of this study are:

1. To assess the feasibility and safety of combined PEX, rituximab, and conventional corticosteroid administrations for the treatment of hospitalized patients with acute IPF exacerbations by monitoring indices of respiratory (PaO2) and cardiovascular function during the treatment interval.
2. To assess the efficacy of combined PEX, rituximab, and conventional corticosteroid administrations for the treatment of hospitalized patients with acute IPF exacerbations on patient survival in comparison to historical controls. Patient survival for this investigation will be defined using the composite outcome of 60 day survival and/or survival to lung transplantation.

Subjects between 18 and 80 years of age who have a confirmed diagnosis of IPF, and meet all the study requirements will be enrolled in this study. A total of 10 subjects of both genders and all ethnic backgrounds with acute IPF exacerbations hospitalized at UPMC will be enrolled in this study.

**STUDY PRINCIPAL INVESTIGATORS:**

**Principal Investigator: Michael Donahoe, MD**

**Co-Principal Investigator: Steven Duncan, MD; Kevin Gibson, MD**

**SECTION 1 – STUDY OBJECTIVE, SPECIFIC AIMS, BACKGROUND, AND SIGNIFICANCE**

- 1. **OBJECTIVE**

The goal of this open-label Phase I/II clinical trial is to assess the feasibility, safety and preliminary comparison of efficacy of combined plasma exchange (PEX), rituximab, and conventional corticosteroid administration on the survival of patients with acute IPF exacerbations in comparison to historical controls.

Heretofore, most patients who develop acute exacerbations of IPF succumb to the lung disease within days to a few weeks. Current, conventional treatment using high, albeit unspecified, doses of corticosteroids seem to have no therapeutic efficacy. Lung transplantation is believed to be an effective treatment for IPF, but many patients with the acute manifestation of this disease who are referred to UPMC die before their lung transplantation evaluations are complete, and/or before a suitable donor lung can be procured.

We have recently found evidence that pathogenic processes leading to IPF progression appear to be mediated by autoantibodies. Very analogous autoimmune responses are also involved in some cases of chronic lung allograft rejection and other acute lung injuries (ALI), including adult respiratory distress syndrome (ARDS) associated with polymyositis and other conventional autoimmune syndromes. As is the case with IPF, neither chronic allograft rejection nor the other autoantibody-mediated ALI syndromes respond to corticosteroids or other simple, nonspecific immunosuppressive agents. However, treatments that specifically reduce autoantibody production (e.g., rituximab) and/or remove pre-existent autoantibodies (e.g., plasma exchange [PEX]) appear to be more efficacious, and have even rescued critically ill patients with severe respiratory failure who did not respond to conventional steroid-based regimens.

We hypothesize that acute IPF is a manifestation of autoantibody-mediated lung injury, and that mechanistically-focused treatments will have better efficacy than nonspecific, current standard medical therapy. We further anticipate that our rituximab + PEX treatment will result in improved short-term mortality of IPF patients with acute exacerbations, enabling many more of them to survive long enough for definitive lung transplantation. As such, the experimental treatment proposed here will be a beneficial “bridge” to “buy time” for these otherwise doomed patients to benefit from curative lung transplantation.

- 1. **SPECIFIC AIMS**

1. To assess the feasibility and safety of combined PEX, rituximab, and conventional corticosteroid administrations for the treatment of hospitalized patients with acute IPF exacerbations by monitoring indices of respiratory (PaO2) and cardiovascular function during the treatment interval.

2. To assess the efficacy of combined PEX, rituximab, and conventional corticosteroid administrations for the treatment of hospitalized patients with acute IPF exacerbations on patient survival in comparison to historical controls. Patient survival for this investigation will be defined using the composite outcome of 60d survival and/or survival to lung transplantation.

**As such, more IPF patients with acute exacerbations will live long enough to have the clinical evaluations necessary for lung transplantation and until suitable donor organs can be procured.**

We hypothesize the combined treatment will ameliorate autoantibody-mediated lung injury and have a demonstrably beneficial effect on patient outcome.

- 1. **BACKGROUND**

Idiopathic pulmonary fibrosis (IPF) is an interstitial lung disease characterized by inflammation and fibrosis of lung parenchyma affecting gas exchange. IPF has a poor prognosis with median survival of approximately 3 years from the time of diagnosis. Although IPF is chronic in nature and usually slowly progressive, some patients may experience an accelerated phase of the disease that has a very poor prognosis, with death occurring in a few days to weeks. Acute IPF exacerbation has been defined as an acute, clinically significant deterioration, without unidentified cause, of respiratory status in patients with underlying IPF. Current criteria for acute IPF exacerbations include a combination of the following that have occurred within a 30 day period: exacerbation of dyspnea and/or evidence of abnormal gas exchange, as defined by a lower partial pressure of arterial oxygen (Pa_O2_)/percentage of inspired oxygen (Fi_O2_) ratio or a decrease in Pa_O2_; new bilateral radiographic opacities; and an absence of infection or another identifiable etiology such as left heart failure, or pulmonary embolism.

The etiology of IPF remains elusive,^1-3^ and the mechanism(s) leading to acute exacerbations of this disease are especially mysterious.^4^ The majority of IPF patients with acute exacerbation are admitted to intensive care units with acute respiratory failure. To date, there is no proven successful treatment for acute IPF exacerbation that has been shown to prolong survival, aside from lung transplantation. Treatment of acute IPF exacerbations traditionally has consisted of high-dose corticosteroids (pulse solumedrol), but unfortunately, many patients do not respond to treatments. During the past year, 42 patients were admitted to UPMC PUH with IPF exacerbations and the hospital mortality rate was approximately 50%. The majority of these patients died awaiting lung transplantation. Accordingly, the development and demonstration of a feasible medical treatment that prolongs short-term patient survival, could act as a bridge to transplantation. This would have a hugely beneficial effect by enabling more of these otherwise doomed patients to have the only currently recognized treatment for this disease process.

Our recent studies, and others,^5-9^ show the presence of aberrant immune responses in IPF patients that support an autoimmune pathogenesis, leading us to posit the central hypothesis of this proposal: Antibody-mediated autoimmunity plays an important role in IPF exacerbations.

In addition to relevant preliminary data, the plausibility of our central hypothesis is also supported by analogies to other antibody-mediated acute lung injuries (ALI). Manifestations of acute IPF exacerbations are very similar to sudden deteriorations of interstitial lung disease among myositis patients with anti-synthetase autoantibodies,^10,11^ and lung transplant recipients with allograft rejection due to anti-donor HLA antibodies.^12^ Autoantibodies against IL-8 (and other autoantigens) have also been implicated in some cases of adult respiratory distress syndrome.^13,14^ These syndromes all share common features of rapid pulmonary deterioration in the absence of other obvious extrinsic processes, as well as histological patterns of diffuse alveolar damage and, notably, nearly invariable refractoriness to corticosteroids.

In contrast, however, *specific treatments to remove or reduce autoantibodies are now a mainstay of therapy for anti-donor HLA antibody-mediated lung transplant rejection,^13^ and have also been effective in allograft recipients and other patients with acute autoantibody-mediated lung diseases who failed prior courses of corticosteroids.^11,12,15^*

The production of IgG antibodies (and autoantibodies) with specificity for protein determinants is the result of coordinated interactions between the three major elements of the adaptive immune system: 1.) HLA molecules; 2.) T-cells; and 3.) B-cells. In brief, highly specific peptide antigens are presented by HLA molecules of antigen presenting cells, including B-cells, to the limited pool of T-cells with antigen receptor specificity for these particular peptide-HLA complexes. In turn, these now antigen-activated “armed effector” T-cells divide, generating sometimes prodigious numbers of clonal daughter progeny that elaborate myriad mediators which directly injure tissues, and/or activate and recruit other immune effectors, including B-cells.^16,17^

With respect to our supportive/preliminary data to follow, all IPF clinical specimens were (and will continue to be) obtained from carefully scrutinized subjects who fulfill consensus ATS/ERS diagnostic criteria.^1^ Diagnoses were prospectively established by expert specialized clinicians blinded to experimental study results, who analyzed all clinical information, including medical histories and physical exams, pulmonary function tests (PFTs), laboratory studies that included serologic tests for conventional autoimmune syndromes, as well as expert rheumatologist evaluations, chest radiographs, and HRCT scans. No IPF study subject had evidence or a history of connective tissue diseases, drug toxicities, or occupational/environmental exposures associated with lung disease. All lung histology from subjects showed usual interstitial pneumonia (UIP) or end-stage fibrotic disease. Specimens from patients with clinical, histological, or microbial evidence of ongoing infections were not analyzed. Laboratory investigators were blinded to subject identities. Mann-Whitney tests were used for comparisons of unpaired continuous or ordered variables, and a Wilcoxon was used for paired tests. Dichotomous variables were compared by chi-square, and odds ratios (OR) and 95% confidence intervals (CI) established by logistic regression. Hazard ratios (HR) and CI were established by proportional hazard. Survival analyses were performed using product-limit, with comparison by log-rank. Data here are depicted as means + SEM and p values are delineated.

Our studies to date have found interrelated abnormalities of all three adaptive immune elements in IPF patients that support the central hypothesis of this proposal:

**1.3.1 HLA Allele Frequency Aberrations in IPF**

Each distinct HLA allele has a restricted motif for binding and presentation of peptide antigens to T-cells.^18^ Hence, HLA haplotype inheritance determines the finite repertoire of antigens that can evoke adaptive immune responses in an individual. Although critical for host defenses, these responses may be deleterious if the antigen is a self-protein (autoantigen), or one that triggers a cross-response to a self-protein.^19-24^ In contrast, individuals lacking these “permissive” HLA alleles do not present the disease-associated antigens, and do not initiate the deleterious response(s). *A concomitant of autoimmune syndromes is the frequent finding that distinct HLA alleles are over-represented among those afflicted with these diseases*.^19-21,25-30^

We found HLA allele DRB*15 is over-represented in IPF patients:^31^ The initial discovery cohort for these HLA characterizations consisted of 79 consecutive IPF patients who had molecular HLA allele determinations prior to lung transplantation, beginning in March 2006. Controls consisted of 196 normal subjects. Analyses are restricted to Caucasians because <5% of the transplant recipients were members of minority groups, and HLA allele frequencies can vary greatly among racial/ethnic subpopulations.^32^

The initial analysis showed the allele prevalence of DRB1*15 (i.e., the proportion of subjects who have either one or two copies of this allele) was 37% among the IPF vs. 23% in the normal cohort (p = 0.02). No other HLA allele was significantly over-represented (data not shown). The DRB1*15 frequency among IPF patients was also greater than in other Caucasian normals,^29,30^ including a very large compilation (n = 6396),^32^ whereas the frequency of this allele in our controls was ~identical to those other reports.

Based on these findings, validation cohorts were prospectively analyzed for DR15B*15 among 35 IPF and 41 controls from the NIH, 20 IPF patients at Inova, Fairfax, VA, and 14 IPF from Stanford. DRB1*15 was determined by RT-PCR sequence specific primer assay. This allele was over-represented in each IPF cohort at all venues, and aggregate prevalence was similar to that of the discovery population (Figure 1). Three IPF patients had relatives who died with lung disease(s). None of these 3 had DRB1*15.

**Figure 1**. Prevalences of DRB1*15 were greater in IPF cohorts compared to normal controls. Numbers within columns denote subject n.

Most IPF HLA studies date from the distant past, before development of accurate molecular methods, full knowledge of HLA Class II alleles, and subject numbers were small.^33-38^ Nevertheless, all but two^34,35^ indicated HLA allele perturbations are present in IPF. The only recent study using molecular techniques reported HLA Class II alleles, including DRB1*01, *04, and *14, were over-represented in a single-cohort Mexican IPF population.^39^ We did not see abnormal frequencies of those alleles in our subjects (data not shown). Conversely, the DRB1*15 frequency among the IPF patients of that study^39^ was much less than in our normal cohort, or other normal populations.^29, 30,32^

**1.3.2 T-cell Abnormalities in IPF**

Our studies, and others, also show T-cells of IPF patients are abnormally activated, have enhanced production of IPF-relevant inflammatory (e.g., TNF-α) and pro-fibrotic mediators (e.g., IL-4, IL-13, TGF-β, etc.), and impaired regulatory (T_reg_) function.^8,9,40-42^ Oligoclonal T-cell proliferations are present in IPF subjects ^8,42^, and no other mechanism can account for these findings other than conventional antigen stimulation.^43^ A protein antigen(s) within IPF lungs drives autologous CD4 T-cell proliferations, which is “Gold Standard” evidence of abnormal T-cell reactivity, since T-cells of normal humans do not overtly react to accessible antigens within their organs.^8^ Highly abnormal proportions of phenotypically distinct T-cells with enhanced cytotoxic and pro-inflammatory function, (e.g., CD4^+^CD28^null^ cells) are present in the circulation and lungs of IPF patients^9^ (see also Figure 2).

**Figure 2**. Flow cytometry gated on CD56^-^CD3^+^CD4^+^ cells isolated from an enzymatically digested IPF lung explant show a large proportion of CD28^null^granzyme B^+^ infiltrating cells.^49^ Cells were >95% viable (per 7AAD exclusion).

These unusual T-cells are end-differentiated daughter progeny of repetitive antigen-driven proliferations, *and a specific feature of chronic immunologic disorders*.^44^ CD4^+^CD28^null^ cells autonomously produce pro-inflammatory cytokines, granzyme B, have shortened telomeres, and lack T_reg_ potential.^9,44,45^ CD4^+^CD28^null^ cells are also relatively refractory to effects of cyclosporine^9^ and steroids (Figure 3A). Significantly, the extent of this CD4 T-cell differentiation is associated with patient outcomes in diverse chronic immunologic disorders, including IPF^9,44-48^ (Figure 3B).

Other T-cell alterations occur with chronic stimulation and repetitive proliferations, including **paradoxical down-regulation** of CD25, CCR7, CD45RO, etc.^9,45,49^ Changes in these markers also correlate with the extent of chronic immune responses, and also have prognostic associations in IPF patients (Figure 3C).

**Figure 3**. **A.)** CD4 T-cells altered by repetitive antigen-driven proliferations (CD4^+^CD28^null^)^41^ are steroid resistant. Circulating IPF CD4 were sorted into CD28^null^ and CD28^+^ subpopulations, stimulated with anti-CD3 antibody, and proliferation measured by BrdU uptake. **B.)** Survival is decreased among IPF patients with greater proportions of CD4^+^CD28^null^ cells (High vs. Low stratifications were by ROC curve).^9^ **C.)** Analyses of other T-cell phenotypic markers that also change with repetitive T-cell proliferations (15) are associated with survival of IPF patients.^49^

**1.3.3 The Role of B-cells in IPF**

B-cells elaborate IgG after receiving “help” from antigen-specific CD4 T-cells.^17^ IPF patients have many B-cell abnormalities, including the presence of pathogenic B-cell aggregates in IPF lungs,^50-52^ as well as intrapulmonary over-expression of immunoglobulin genes.^53^ Antibody-antigen complexes are also present in sera and bronchoalveolar lavage fluid (BALF) of these patients.^54,55^ Diverse autoantibodies are found in IPF cohorts^5-8,55-59^ and, by using relatively sensitive methods, we have shown self-reactive IgG autoantibodies are present in most of these patients.^8^

IgG autoantibodies exert deleterious effects by numerous mechanisms. Engagements with cell surface ligands or intracellular antigens (*antibodies can gain entry into cells via lipid rafts and other mechanisms^60^*) can result in disordered production of important mediators or other dysfunctions.^59,61,62^ IgG autoantibodies can also directly induce target cell apoptosis by antibody-dependent cell-cytotoxicity (ADCC).^63^ Immune (antibody-antigen) complex deposits in tissue activate complement, causing cytotoxicity, and activation and recruitments of neutrophils that generate ROS intermediaries.^64^ Autoantibodies from IPF patients in particular have been shown to increase epithelial cell production of TGF-^59^ and kill endothelial cells^5^. *Acute IPF exacerbations have been previously shown to be associated with specific autoantibody responses to IL-1*α *and annexin 1.^67^* We also recently found anti-Hsp70 autoantibodies in IPF patients are associated with impending disease progression (below).

**1.3.4 IPF Autoantigen Discovery**

We had previously shown most IPF patients have circulating IgG autoantibodies against diverse cell lysate proteins.^8^ The presence of one in particular with avidity for a cryptic ~70-80 kDa autoantigen was associated with pulmonary function decrements (unpublished data). To identify these autoantigens, cell lysate proteins were immunoprecipitated, using pooled IgG from IPF patients. Lysates were first run thru normal IgG bound to protein A (to minimize nonspecific binding). Eluants were then applied to columns of IPF IgG covalently bound to protein A, washed, the IgG-bound cell proteins (putative autoantigens) were eluted by acidification and electrophoresed on 2-D gels (Figure 4). Individual proteins were spot picked, trypsin digested, and sequenced by mass spectrometry (MALDI-TOF/TOF). Three of these assays have been performed to date.

Proteins in the 70-80 kDa range were initially targeted (given indications of a pulmonary function correlate). Hsp70 was identified in all three replicate preparations (Figure 4). Hsp70 also seemed to have plausibility as an autoantigen, given analogous findings in other autoimmune disorders.^65-68^

**Figure 4**. Two-dimensional (2-D) gel electrophoresis of cell lysate proteins immunoprecipitated by IPF patient IgG (Coomassie stain). Circles denote proteins.

**1.3.5 Anti-Hsp 70 Autoantibodies in IPF Patients**

Plasma specimens were obtained by centrifugation of anti-coagulated blood, and screened for autoantibodies to recombinant Hsp70 (rHsp70) by immunoblots. Immunoblot results are dichotomous (positive or negative) and highly specific. rHsp70 (250 ng) was loaded onto gels and electrophoresed. Individual membrane lanes were cut out and incubated with 1:10 dilutions of individual plasmas. Chicken anti-human IgG-HRP (1:8000) was used as the secondary antibody. Characteristics of the IPF subjects are detailed in Table 1. Healthy controls were comparable in terms of age (64 + 2 y.o.) and gender distribution (65% males). Results are depicted in Figure 5. Antibodies to Hsp70 were not detected in any of 20 asthmatics and in only one of 26 GOLD 1 COPD patients (3.8%) tested to date. **Note**: the anti-Hsp70 detected here were IgG, and not the IgM isotype associated with benign “natural” autoantibodies.^69^

**Figure 5**. Anti-Hsp70 autoantibodies were more prevalent in IPF patients compared to normal controls. Numbers within columns denote subject n.

**1.3.6 Hsp70 in IPF Lung Specimens**

To be biologically plausible, the antigen of an autoimmune response needs to be present in the target organ. Hsp70 was assayed in BALF and water soluble extracts of lung explants- previously shown to be a rich source of IPF T-cell antigens,^8^ by immunoblots using anti-human Hsp70 monoclonal antibodies (Stressgen). Protein concentrations were measured by bicinchoninic acid assay and equal amounts applied to each lane. Hsp70 was present in all IPF pulmonary specimens, but was less often seen in the preparations from normal lung explants (Figure 6).

**Figure 6**. Representative immunoblots used to detect anti-Hsp70 IgG in plasma (Row 1), and Hsp70 antigen in lung explant extracts (n = 6) and BALF (n = 5).

**1.3.7 IPF Lung Immunohistochemistry (IHC)**

IHC was used to localize Hsp70 expression in IPF lung explants. Hsp70 was present in all six IPF lungs (*per expert pathologist interpretation*) in distal airway epithelium, alveolar epithelial cells, alveolar macrophages and some endothelial cells (Figure 7). Hsp70 was much less frequently expressed in normal lung IHC sections (data not shown-see also Figure 6).

**1.3.8 Intrapulmonary Immune Complexes and Complement (C3) in IPF Lungs**:

Antibody-antigen immune complexes and fixed complement in tissues are both features of autoantibody-mediated disease processes. Methods for *in situ* IHC detection of antibody-antigen (immune) complexes and fixed complement have been described previously.^63^ We previously showed these deposits are not present in normal lungs,^63^ and those results were confirmed using 6 “new” normal lung specimens (data not shown). In contrast, IgG complexes were present in 5/6 IPF lung explants removed during therapeutic transplantations (these specimens are distinct from the Figure 6 specimens) and C3 was evident in three (Figure 7).

**Figure 7**. IHC of IPF Lungs. Top Row: stable, severe end-stage IPF lung explant removed during therapeutic transplantation: A) Hsp70 expression (20x); B) IgG immune complexes (40x); C) C3 deposits (40X); D) isotype control (20x). Bottom Row: Lungs from patients who died from respiratory failure due to acute IPF exacerbations showed equally or more prominent E.) Hsp 70 expression: F.) Immune complex deposition: and G.) Fixed C3. H.) is the isotype control for this lung.


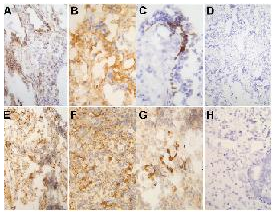


**1.3.9 Clinical Correlates of Hsp70 Autoreactivity in IPF Patients**

PFT and outcome measures were compared between the IPF patients with anti-Hsp70 autoantibodies (Antibody Pos) and those who did not have these autoantibodies (Antibody Neg). There were no significant intergroup differences at the time of their specimen acquisitions (Table 1). There were, however, greater subsequent decrements of pulmonary function in Antibody Pos patients during the next few months (Figure 8).

**Figure 8**. IPF patients with anti-Hsp70 autoantibodies had greater decrements of %predicted FVC and a trend for decreased %predicted DLCO. PFTs were measured 6 + 1 month after the plasma acquisitions.

One-year mortality was also greater among the Antibody Pos and this survival difference was evident within a few weeks (Figure 9). Respiratory failure accounted for 78% of deaths among Antibody Pos and 55% in Antibody Neg. Aside from a fatal pulmonary embolus among the latter, other deaths could not be specifically attributed.

**Figure 9**. Survival of Antibody Pos subjects was significantly decreased compared to the IPF patients who did not have this autoantibody.

*Post hoc* analyses limited to those patients not taking immuno-suppressants (Table 1) revealed Hsp70 autoantibodies were present in 19% (p = 0.018 vs. controls). One-year survival was 34% among un-medicated Antibody Pos and 78% among un-medicated Antibody Neg patients (p = 0.0008).

|  | **n** | **Age (yr)** | **%male** | **FVC%p** | **DLCO%p** | **%Smoke** | **%Meds** |
| --- | --- | --- | --- | --- | --- | --- | --- |
| **Autoantibody Positive** | 21 | 71 + 2 | 81 | 58 + 3 | 43 + 4 | 62 | 19 |
| **Autoantibody Negative** | 67 | 69 + 1 | 70 | 63 + 3 | 48 + 3 | 45 | 22 |

**Table 1.** Demographics of IPF subjects with and without anti-Hsp70 autoantibodies. %p = percentages of predicted values; %smoke = percentages with >5 pack year histories; %meds denote percentages of subjects taking single agents or combinations of prednisone (5-20 mg/day), azathioprine, interferon-γ, mycophenolate, or tacrolimus. None of the differences were significant. This table needs to be reformated

**1.3.10 Hsp70 Autoreactivity and HLA-DRB1*15 in IPF Patients**.

Autoantigen-specific immune responses often associate with particular HLA alleles.^19-21,25-30^ We determined the presence of DRB1*15 among the 88 IPF patients who had anti-Hsp70 assays. DRB1*15 was significantly more prevalent in those with anti-Hsp70 autoreactivity (Figure 10). DRB1*15 is also a frequently over-represented HLA allele in many immune diseases,^25-30^ including Goodpasture’s syndrome and SLE, both of which are also autoantibody-mediated diseases.^15,20,21^

**Figure 10**. DRB1*15 is over-represented in IPF patients with anti-Hsp70 auto-antibodies.

- - 1. **Specificity of Anti-Hsp70 Autoantibodies for IPF Clinical Associations**

Of many potential autoantigens tentatively identified (Fig. 4), the only other one extensively tested to date (aside from Hsp70) is glucose regulated protein 78 (Grp78), a 78 kDa heat shock protein with extensive homology to Hsp70.^70^ However, there is little concordance between anti-Hsp70 and anti-Grp78 autoantibody prevalences in IPF patients (Figure 11). Moreover, we have seen no associations (or even trends) between the presence or absence of anti-Grp78 IgG and HLA frequencies or pulmonary function nor patient survival (data not shown). These findings suggest that even though some autoantibodies may be generated nonspecifically in IPF (in response to chronic inflammation?), at least some others, e.g., anti-Hsp70 are associated with disease manifestations (Figs. 8 and 9). *Concurrent findings of both disease-relevant and seemingly epiphenomenal autoantibodies are a common feature of autoimmune syndromes*.^20,21^

**Figure 11.** Anti-Grp78 IgG autoantibodies are also frequent in IPF, but have little concordance with anti-Hsp70 responses. Numbers in wedges denote percentages of the total IPF population (n = 88).

- - 1. **Genetic Polymorphisms and IPF Patient Outcomes:**

**These particular preliminary data in this section are proprietary, and as of yet unpublished. They were included in the protocol but were never intended for wide or uncontrolled dissemination or publication. They have no bearing on the submitted manuscript and are omitted here.**

**1.3.13 IPF T-cell Reactivity to Hsp70**

Finding T-cells from patients are also reactive to the humoral self-antigen is HIGHLY INDICATIVE of an antigen-specific pathogenic autoimmune response.^20,21^ Indeed, it is difficult to envision a benign, disease-irrelevant scenario wherein autologous CD4 T-cells proliferate and elaborate cytokines in response to an accessible self-protein (Figs. 6,7), since these T-cell responses are typically highly injurious.^16^ *It bears emphasis that T-cells from normal individuals do not react to assessable self proteins*. Functional assays show Hsp70 is an autoantigen for CD4 T-cells of most IPF patients (Figure 13).

**Figure 13**. (**A**) Proliferation, per BrdU incorporation in CD4 T-cells from IPF patients, was greatest in co-cultures supplemented with Hsp70, compared to those with elastin split products,^71^ recombinant kexin (a *Pneumocystis* cell wall antigen) or tetanus toxoid (TdT). Specific proliferation is calculated as proliferation in experimental protein-added cultures *minus* control (unstimulated) cultures (see methods in ref. 8). Results are shown for 5 day cultures with 1 ug/ml of added protein, with no consistent difference noted for concentrations ranging from 0.1-10 μg/ml. P values represent Wilcoxon comparisons of experimental vs. control values. Specific proliferation of positive controls (stimulated with anti-CD3) was 39.1+4.6 (p<0.0001). (**B**) Most IPF patients had positive proliferative responses to Hsp70. (**C**) We have shown abnormally increased proportions of IPF CD4 T-cells produce IL-4.^8,49^ Greater proportions of IPF CD4 T-cells produced IL-4, a pro-fibrogenic cytokine, in co-cultures with Hsp70. CD4 T-cells from age, gender, and smoking-matched normal controls do not react to Hsp70 (data not shown).

**1.3.14. Other Ongoing Studies**

Other ongoing studies include longitudinal study of Hsp70 autoreactivity in IPF patients. So far, 5 of 6 IPF who were initially anti-Hsp70 IgG negative, but died >12 months later, were shown on serial testing to develop anti-Hsp70 IgG a few weeks prior to their deaths. Disease controls (e.g, severe COPD) will also be surveyed, as well as determinations of immunologic-clinical associations for other putative autoantigens discovered by IP assays (Fig. 4). Finally, we have isolated “pure” anti-Hsp IgG from patient specimens (using rHsp70 bound to agarose), and will show specific pathogenicity of this preparation with human primary pulmonary cells *in vitro*, as we did with COPD autoantibodies,^63^ and try to fulfill Koch’s postulates in an animal model.^72^

- - 1. **Supportive Data Summary**.

Findings presented here, showing the presence of anti-Hsp70 IgG self-reactivity (Fig. 5), an association with a distinct HLA allele (Fig. 10), presences of the autoantigen and immunopathogenic processes in the diseased organ (Figs. 6,7), clinical correlates of autoreactivity (Figs. 8,9), an autoantigen SNP associated with survival (Fig. 12), and T-cell autoreactivity (Figure 13), fulfill conventional criteria of disease-relevant autoimmunity.^19-21^

Nonetheless, we are not proposing that autoreactivity to Hsp70 or any other particular autoantigen discovered so far is the “CAUSE” of IPF. Moreover, we make no claims that anti-Hsp 70 autoreactivity is STRICKLY SPECIFIC for IPF (it is not^65-68^). Along these lines, although antinuclear antibodies (ANA) are an important tool for SLE diagnosis and treatment, these serologic tests are also positive in other rheumatologic diseases, chronic active hepatitis, viral infections, some drug reactions, and some IPF patients. The cumulative weight of interrelated findings hereand elsewhere^5-9,42,54-59^ are as compelling as the evidence for most conventional autoimmune syndromes,^19-21^ and support the potential role of autoimmunity in IPF progression.

One plausible explanation of these observations, which is also a paradigm for the pathogenesis of other autoimmune (and many fibrotic)^73^ syndromes, can be outlined: An environmental agent (microbial or viral infection, or inhaled antigen[s]?) causes an initial lung injury and triggers an adaptive immune response. Activated, antigen-specific CD4 T-cells, in turn, fuel an inflammatory cascade with myriad down-stream injuries.^16^ With repetitive stimulation, these antigen-activated T-cells undergo clonal expansions^8,43^ and develop heightened, dysregulated pathogenic potential.^9,44-48^ Although the initial immune conflagration may have been appropriately targeted against the foreign antigen, the response becomes redirected by epitope spread^23^ or mimicry^24^ to now also include inappropriate self-antigen targets, and production of autoreactive IgG^17^, at least among individuals who have permissive genetic backgrounds (e.g., particular HLA alleles^19-21,25-30^). Once initiated, autoimmune responses tend to be self-perpetuating and may be progressive, despite removal or cessation of the inciting processes, since the target self-antigens are continually renewed. Ongoing inflammation can itself cause increased expression of self-antigen in cases where these proteins are stress response molecules (e.g., Hsp70),^65-68^ creating a positive feedback loop that could accelerate disease progression (*Could this explain the unremitting nature of IPF and acute exacerbations in some of these patients?*). Moreover, autoantibody injuries are often unresponsive to corticosteroids^11,12,15,74-77, and many others^ (*Could this explain IPF resistance to nonspecific steroid regimens?* ^1-4^).

- 1. **SIGNIFICANCE**

The proposed use of combined plasma exchange (PEX), Rituximab, and corticosteroids administration is an innovative approach that we hypothesize will have a beneficial effect on the survival of patients with acute IPF exacerbation for the following reasons:

1) We hypothesize the combined therapy that rapidly removes autoantibodies (PEX) and minimizes their subsequent production (rituximab), will have a beneficial effect on 60-day patient survival.

2) This Phase I/II clinical trial will also enable systemic collection of otherwise rare patient specimens for unprecedented, laboratory-based Ancillary Studies.

3) Results of the proposed studies could also have relevance for the larger population of IPF patients with more typical slowly deteriorating lung function.

4) The trial has the potential to profoundly affect current paradigms and treatment approaches to this disease manifestation, and could ultimately be broadly relevant to typical IPF patients with more slowly progressive disease.

*5)* The research proposed here has the potential to contribute to a major paradigm shift in concepts of IPF pathogenesis and clinical management, and could perhaps even have implications for treatment of other autoantibody-mediated ALI syndromes.

**SECTION 2 – RESEARCH DESIGN AND METHODS**

**2.1 CLASSIFICATION AND METHODOLOGICAL DESIGN**

This is a prospective, open-label Phase II, non-randomized clinical trial to assess the feasibility and safety of combined plasma exchange (PEX), rituximab, and conventional corticosteroid administration in patients with acute IPF exacerbations.

**2.2 DETAILED DESCRIPTION OF RESEARCH ACTIVITIES**

**2.2.1 Study Design and Procedures**

The proposed combined treatment of plasma exchange (PEX), rituximab, and corticosteroids is routinely provided for a specific subset of patients with a unique form of autoimmune interstitial lung disease, called anti-synthetase syndrome or anti Jo-1 antibody syndrome at UPMC PUH. In this proposal, we plan to extend this treatment to the more general IPF exacerbation patient population which currently is most commonly treated with high dose corticosteroids or pulse solumedrol alone. All clinical and laboratory testing will be considered part of routine medical care for patients with no therapeutic options and a very high recognized mortality rate. In addition to routine medical care, specific T-cell and B-cell assays will be conducted in the laboratory of a Co-PI (Dr. Duncan).

After hospital admission for acute IPF exacerbation (and after informed consent is obtained), subjects will receive the standard steroid treatment followed by initiation of the PEX and rituximab regimens. These latter two agents will require insertion of a dialysis/apheresis catheter into a central vein.

Standard Steroid Treatment: One gm of methylprednisolone i.v., on day 0, followed by 40 mg/day i.v. on days 1-4, and days 6-12 (or the p.o. prednisone equivalent). Methylprednisolone 100 mg i.v. will be administered on days 5 and 13. Steroid doses will then be 20 mg methylprednisolone i.v. (or p.o. prednisone equivalent) from days 14-28, and then reduced thereafter at the discretion of the principle investigator at each site. There are no published data that show efficacy of this agent at these doses. Nonetheless, steroid treatments (with highly varied doses and durations) are currently the standard of care for IPF patients with acute exacerbations.^4^ The high initial dose is intended to optimize early lympholysis. Administration of methylprednisolone 100 mg i.v. on days 5 and 13 will standardize steroid treatments among the two trial arms, as these agents are recommended prior to rituximab infusions.^78^ Steroid doses < 20 mg methylprednisolone i.v. (or p.o. prednisone equivalent) from days 14-28 are important to establish as this dose is an appropriate cutoff for consideration of lung transplantation.

Plasma exchange (PEX) will consist of 1.5x estimated plasma volume exchanges for 3 successive days (0, 1,2) and then, after a one day interval to enable equilibration of autoantibodies sequestered in tissues, two more daily treatments on days 4 and 5. It is anticipated that fluid replacement with 5% albumin:normal saline (3:1 ratio), with a net fluid balance of 95-100%, will be possible in nearly all patients for the first two treatments. In the event a subject’s INR is >1.5 at the initiation of a PEX treatment, as may occur on the third or later treatment, the albumin:saline replacement will be partially supplanted with fresh frozen plasma (FFP) (anticipated to be <10 U/treatment) to maintain INR <1.6. The dialysis catheter will be removed after completion of the 5^th^ and final PEX.

Rituximab: One gm i.v. will be administered on day 5 (after completion of the last PEX) and day 13. This regimen is adopted from the protocol of a recent large myositis trial at UPMC and other sites (NIHNO1 AR42273) and was well tolerated. Subjects will be treated with acetaminophen, antihistamines and methylprednisolone 100 mg i.v. prior to administration of rituximab to obviate reactions.^75^

The study design and flowchart is outlined in the table below (Appendix 1).

| Combined Treatment | **Days on Interventions** | | | | | | | | | |
| --- | --- | --- | --- | --- | --- | --- | --- | --- | --- | --- |
|  | **0** | **1** | **2** | **3** | **4** | **5** | **6-12** | **13** | **14-28** | **> 28** |
| Steroids | 1 gm /day, IV | 40 mg/day, IV  or oral Prednisone equivalent | | | | 100 mg  /day, IV | 40 mg/day, IV  or oral Prednisone equivalent | 100 mg  /day, IV | 20 mg/day, IV  or oral Prednisone equivalent | Tapering thereafter at the discretion of the treating physician |
| Plasma Exchange | 1.5x estimated plasma volume exchange/day | | | NA | 1.5x estimated plasma volume exchange/day | | NA | | | |
| Rituximab | NA | | | | | 1gm  /day, IV | NA | 1 gm  /day, IV | NA | |

**2.2.2 Data Collection**

Routine clinical care for seriously ill patients with respiratory insufficiency will be followed as outlined by the primary physician. The study will log the results of monitoring labs consisting of complete blood count (CBC), electrolytes, glucose, BUN, serum creatinine, ionized calcium, magnesium, phosphorus, INR, albumin, liver function tests including of total bilirubin, aspartate alanine transaminase (ALT), and aspartate glutamine transaminase (AST), and complement studies (C3, C4, CH50). These laboratory tests will be logged from the closest available value (+/- 48 hours) on Days 0, 7, 14, 28 or at hospital discharge if < 28 days. In addition to the time points specified above, INR and Ionized calcium will be performed daily during the days when subjects undergoing PEX.

An extra 16 ml of blood needed for T-cell and B-cell studies for research purposes will be drawn at times corresponding to routine blood draws for clinical lab tests. These assays will be carried out in the laboratories of Dr. Steven Duncan.

During hospitalization, subjects will be visited by study personnel within 24 hours after enrollment, and on Days, 7, 14, 28 (or on the day of discharge). A variance of two days will be allowed in the usual follow-up schedule. However, the 24 hour visit and discharge visit should occur on the specified day. In addition, a daily progress note will be made by the treating physician, and placed in the inpatient chart and research subject binder as a source document.

At these visits (Days 0, 7, 14, 28 or at hospital discharge), the following information will be collected and entered into to the study case report forms (CRFs). All clinical and laboratory data are part of routine clinical care and will be collected from medical records.

1. Demographics, physician names and contact information, general medical and surgical histories, co-morbidities, concurrent medications, and allergies.
2. Recording of vital signs and medication regimen.
3. Review of medication changes
4. Brief assessment of symptoms and documentation of any adverse effects.
5. Recording of laboratory and clinical testing results.

A telephone contact will occur on Day 60 for post treatment survival surveillance. If the subject remains inpatient, the assessment may be completed in the hospital setting. Subjects will be contacted by the study personnel to assess for symptoms potentially related to adverse effects of medications, review of medication changes, and interval medical or surgical histories including emergency or physician office visits. A variance of 3 days will be allowed for the telephone contact to facilitate scheduling or to account for weekends or holidays.

The schedule for patient assessment is outlined as follows (Appendix 2).

Day 0 (Enrollment):

- Informed Consent
- History and physical exam to include vital signs and blood pressure.
- Laboratory evaluations including complete blood count (CBC), electrolytes, glucose, BUN, serum creatinine, calcium, magnesium, phosphorus, albumin, liver function tests, and INR.
- Serology for hepatitis B (HbsAg).
- Assessment of PaO2.
- Complement studies of C3, C4, CH50.
- Assessment of peripheral blood T-cell and B-cell assays .

Day 7:

- Brief history and physical exam to include vital signs and blood pressure.
- Laboratory evaluations including complete blood count (CBC), electrolytes, glucose, BUN, serum creatinine, calcium, magnesium, phosphorus, albumin, liver function tests, and INR.
- Assessment of PaO2.
- Complement studies of C3, C4, CH50.
- Assessment of peripheral blood T-cell and B-cell assays.

Day 14:

- Brief history and physical exam to include vital signs and blood pressure.
- Laboratory evaluations including complete blood count (CBC), electrolytes, glucose, BUN, serum creatinine, calcium, magnesium, phosphorus, albumin, liver function tests, and INR.
- Assessment of PaO2.
- Complement studies of C3, C4, CH50. See above
- Assessment of peripheral blood T-cell and B-cell assays see above

Day 28 (or at hospital discharge if < 28 days):

- Brief history and physical exam to include vital signs and blood pressure.
- Laboratory evaluations including complete blood count (CBC), electrolytes, glucose, BUN, serum creatinine, calcium, magnesium, phosphorus, albumin, liver function tests, and INR.
- Assessment of PaO2.
- Complement studies of C3, C4, CH50.
- Assessment of peripheral blood T-cell and B-cell assays.

Day 60:

- Brief assessment of symptoms and documentation of adverse events, and a review of medical or surgical histories.

**2.3 STUDY ENDPOINTS**

**2.3.1. The Primary End-Points**

The primary outcome measures for this study are the feasibility and safety of the regimen in patients with IPF exacerbations. Safety will be assessed by monitoring three variables of cardiopulmonary function:

- Respiratory deterioration defined as P_a_O_2_ on days 0, 3, 5, 7, 14, 28 (if still hospitalized) while breathing 100% F_i_O_2_ for >20 minutes by either endotracheal tube (ETT) or nonrebreathing face mask if not intubated (face masks and O_2_ delivery systems for nonintubated patients will be standardized).
- Hemodynamic deterioration defined as need for initiation of vasopressor agents, inotropes, or intravenous diuretics.

**2.3.2. Secondary End-Points**

The secondary outcome measures a composite outcome defined as survival to 60 days or survival to transplantation at any time post therapy. Comparison will be made to historical hospital controls who had IPF exacerbations.

**2.4 STATISTICAL APPROACH**

**2.4.1 Sample Size and Power**

This is an exploratory observational trial to demonstrate feasibility and the statistical power analysis for the sample size is not appropriate. The sample size of 10 for this clinical trial is based upon: 1) exposing a minimum number of patients to potential risks of the experimental treatment while still obtaining useful information; 2) the intent to collect detailed and comprehensive data in each subject to provide comprehensive information relevant for design of a future larger trial, and also provide adequate specimens for the Ancillary Studies. This is an exploratory Phase I/II clinical trial focused on establishing proof-of-concept, and providing access to a novel treatment regimen in patients with a very high risk of hospital mortality.

Given the nature of this initial, exploratory and uncontrolled clinical trial, definitive power calculations are speculative. Nonetheless, high efficacy of the experimental treatment (as we anticipate) could produce evidence of same, given the usual high mortality of historical control patients. We are planning a study with 10 experimental subjects and 100 historical control subjects. A conservative estimate of survival among historical controls is 50%. If our treatment results in 90% survival, we will be able to reject the null hypothesis that the failure rates for experimental and control subjects are equal with probability (power) 0.6.

**2.4.2 Statistical Analysis**

The data analysis for the primary aim begins by describing the sociodemographic, and medical characteristics of the population at baseline. Descriptive statistics, including measures of central tendency and dispersion, will be computed for continuous data (e.g., age). Frequency distributions will be estimated for categorical data (e.g., gender). Mixed effects regression models will be used to assess the treatment effects on gas exchange. Main effects will include treatment, intubation status, time, as well as a treatment by time interaction and random effects for slope and intercept. Additional main effects may include those baseline characteristics not balanced by random assignment, if any are identified. A linear regression model will be used to assess the effect of treatment on the number of ventilation-free days. As with the mixed effects model, main effects will include treatment, intubation status and characteristics not balanced by random assignment, if any are identified. For both the mixed effects model and the linear regression model, the model assumptions (e.g., normality of residuals) will be investigated. If the assumptions are violated, transformations will be investigated. If adequate transformations cannot be identified, nonparametric approaches will be utilized.

Treatment-emergent adverse events are defined as any new event reported after enrollment, or any event that is worse in severity than at any time during the baseline period. Intergroup differences in percentages of treatment-emergent adverse events will be assessed using Fisher's exact test or chi-square test.

**SECTION 3 – HUMAN SUBJECTS**

**3.1 SUBJECT POPULATION**

Subjects for this investigation will be recruited without regard to gender, race, or ethnic background. The anticipated age range for the study population will be >18 years old based upon the target disease population. The study population will be recruited from the inpatient populations at UPMC.

Ten adult subjects of both genders and all ethnic backgrounds with acute IPF exacerbations hospitalized will be eligible for enrollment. All subjects must provide written informed consent prior to participation. Surrogate consent will be obtained for subjects on mechanical ventilation, until the subjects are able to confirm the consent process when they are able to communicate. Based on the referral populations of UPMC, we expect ~40% of eligible subjects will be women and ~10% will be non-Caucasian.

We should be able to achieve these recruitment goals, as the number of potentially eligible patients is far greater than these projected numbers (42 IPF exacerbations at UPMC in the past fiscal year). We anticipate too that nearly all eligible subjects (or their surrogates) will likely elect to participate in this trial, given the poor response to current treatment.^4^ We expect to enroll the proposed study population within one calendar year.

**3.1.1 Inclusion of Women and Minority**

Women who meet the inclusion criteria, and have none of the exclusion criteria, will be enrolled without restriction as dictated by the study protocols. Because of the use of a study medication, woman of child bearing potential must meet specialized inclusion/exclusion criteria to minimize this risk. We will make efforts to enroll participants in this research in a distribution which mirrors the study population of the Pittsburgh area.

**3.1.2 Inclusion of Children**

This investigation will not enroll children based upon to target disease population.

**3.2 INCLUSION CRITERIA**

The inclusion criteria have been selected to isolate a patient population with advanced idiopathic pulmonary fibrosis with a disease exacerbation. The exclusion criteria are selected to not enroll patients with an alternative cause for a respiratory decompensation (ie infection, pulmonary embolism) and to exclude patients with increased risk for the associated intervention (corticosteroids, plasmapheresis and ritiximab). Patients with a coagulopathy are excluded to minimize the risk of catheter insertion associated with the intervention.

1) A diagnosis of idiopathic pulmonary fibrosis that fulfills American Thoracic Society Consensus Criteria.^1*^

2) Unexplained worsening or development of dyspnea or hypoxemia within 30 days leading to the current hospitalization.

3) Radiographic imaging showing ground-glass abnormality and/or consolidation superimposed on a background of reticular or honeycomb pattern consistent with UIP.

4) Intent on the part of the treating physician to use high dose steroid therapy as a therapeutic effort to treat a diagnosis of acute IPF exacerbation.

In addition to strict adherence to current consensus criteria,^1^ IPF patients have serologic determinations of antinuclear antibody (ANA), rheumatoid factor (RF), anti-glomerular basement membrane antibody (AGBM), and anti-nuclear cytoplasmic antibodies (p-ANCA and c-ANCA) as standard of care diagnostic tests. Results of these serologic tests will be retrospectively used to substantiate the absence of confounding autoimmune syndromes in trial subjects, with implications for final data analyses and reporting.

**3.3 EXCLUSION CRITERIA**

1. Diagnosis of documented infection based upon clinical evaluation and microbial testing.
2. Diagnosis of thromboembolic disease by clinical assessment.
3. Diagnosis of an additional etiology for ALI/ARDS based upon clinical assessment to include sepsis, aspiration, trauma, inhalational injury, acute pancreatitis, drug toxicity, blood product transfusion reaction, or stem cell transplantation.
4. Diagnosis of congestive heart failure that accounts for the hypoxemia.
5. Presence of active hepatitis B infection.
6. Coagulopathy defined as an INR > 1.8, PTT > 2 x control, and platelet count < 50K.
7. Hyperosmolar state or diabetic ketoacidosis to suggest uncontrolled diabetes mellitus or uncontrolled hypertension (systolic BP > 160 mm Hg and diastolic BP > 100 mm Hg) which would contraindicated the use of corticosteroids.
8. Hemodynamic instability defined as a vasopressor requirement which would contraindicate the use of plasmapheresis.
9. History of reaction to blood products, murine-derived products, or prior exposures to human-murine chimeric antibodies,
10. History of malignancy.
11. Inability or unwillingness to accept a blood transfusion.
12. Inability or unwillingness to complete post- treatment surveillance for 60 days.
13. Diagnosis of major comorbidities expected to interfere with subjects study participation for 60 days.

**SECTION 4 – RECRUITMENT AND INFORMED CONSENT PROCEDURES**

**4.1 RECRUIMENT METHODS**

Participants will be recruited from the inpatient population at UPMC. Potential research subjects will be first identified by the study investigators, who are also the primary care physician/clinical care team. The clinician investigators will discuss the research project with the potential subjects or their legally authorized representatives as appropriate.

**4.2 INFORMED CONSENT PROCEDURES**

The consent process will begin via one of two possible pathways:

1) Referral of the prospective participant to the investigators/research coordinator by a physician who has knowledge of the proposed research, and obtains patient/surrogate consent for the research team to approach the patient/surrogate.

2) Individuals who have provided signed IRB-approved HIPPA compliant consent for participation in clinical trial research registries.

Prior to performing any of the study procedures the subjects must provide informed consent. The information about this study will be given to subjects in language understandable to subjects. Only physician investigators will present the study to the potential subjects. The physician investigator will verbally present a general outline of the research plan, including inclusion and exclusion criteria, to the prospective participant. The consent form, outlining the design of the study, will include the risks and benefits of participating, and will be reviewed and the investigator will answer any questions. Prospective participants may take as much time as required to make an informed decision. Written informed consent will be obtained from each participant prior to performing any research study procedures.

In addition, older potential study participants whose competency to consent is in question will be tested for sufficient comprehension and recall of the information presented. Prospective subjects who do not remember the important facts about participation in the research study after repeated testing will not be included in the study. The investigators will also assess whether a participant understands experimental procedures over time, including assessment throughout the full duration of participation in the study.

Proxy consent: Given the nature of the study population, which frequently requires mechanical ventilation with sedation, surrogate consent, will occur in a proportion of the study population. This will remain a valid consent until the patient is fully alert, and aware, and can provide a second consent to continue participation in the study.

**Criteria:** If subjects are able to communicate and follow simple instructions on mechanical ventilation, they will be asked to review the protocol and provide consent for participation. Subjects who are unable to demonstrate appropriate orientation to time, person and place as well as an acknowledgement of an understanding of the study design and potential risks, will be deemed unable to provide informed consent.

**Determination of Proxy:** When subjects are unable to provide informed consent, proxy consent will be obtained from the patient’s previously defined surrogate for medical decision-making. If no such surrogate exists, then the subject’s “next of kin” (spouse, children, and siblings, in that order) will be approached, consistent with UPMC hospital policy for obtaining consent for other medical procedures. Subjects judged to be “mentally incapacitated” will be allowed to participate in this study only after consent is obtained from a legal representative authorized to provide consent for research participation.

**Assent:** Once the subject who is incapacitated is able to provide informed consent, this will then be obtained. For mentally incapacitated subjects, assent will be obtained once the patient is extubated and able to communicate verbally. The patient or the surrogate, as identified in the manner outlined here, will sign the consent form prior to enrollment of the subject in the study.

**SECTION 5 – POTENTIAL RISKS AND BENEFITS**

**5.1 POTENTIAL RISKS**

**5.1.1 General Risks of Study Protocol and Procedures**

The potential subject risks specifically related to the study protocol procedures could include:

Venipuncture:

The majority of subjects in this investigation will have existing access for obtaining blood specimen so no venipuncture will be required. For the unlikely subject without vascular access, the risks of the venipuncture are mild and include temporary, minor discomfort which is likely (>25% of the time), bruising which is likely, and which may last for several days, and rarely (<1% of the time) infection, bleeding and phlebitis.

Loss of confidentiality:

The risk of loss of confidentiality with respect to data is rare.

**5.1.2 Potential Risks of Experimental Intervention**

Insertion of a central venous catheter for plasmapheresis:

Complications of this procedure include bleeding at the insertion site, pneumothorax, catheter-associated blood clots and infections (local and systemic). The complication rate of central vein catheters, inserted by physicians of variable experience, has been reported to be 15%.80

Plasmapheresis: The most common adverse effect is a drop in blood pressure associated with symptoms of dizziness or light-headedness79. Unusual complications include extracorporeal coagulation. The addition of citrate to the infusion solutions can lead to hypocalcemia with associated peri-oral tingling, numbness and more rarely muscle irritability, cardiac arrhythmias, and seizures.

Rare complications are infections by blood products (fresh frozen plasma (FFP)), transfusion reaction to FFP, an allergic reaction to the replacement solutions or sterilizing agents for the tubing, and adverse effects of immunosuppression produced by the associated hypogammaglobulinemia. Medications dosing may also need to be adjusted in patients being treated with plasmapheresis as certain medications can be removed by the procedure.

Rituximab:

The most common adverse effects (>10%) with rituximab from available clinical trials include^74,78^:

General: Fever (5% to 53%), chills (3% to 33%), headache (19%), pain (12%)

Dermatologic: Rash (1-15%), pruritus (5% to 14%), angioedema (1-11%)

Gastrointestinal: Nausea (8% to 23%), abdominal pain (2% to 14%)

Hematologic: Cytopenias (2-49%)

Neuromuscular & skeletal: Weakness (2% to 26%)

Respiratory: Cough (13%), rhinitis (3% to 12%)

Infusion Related: angioedema, bronchospasm, chills, dizziness, fever, headache, hyper-/hypotension, myalgia, nausea, pruritus, rash, rigors, urticaria, and vomiting; reactions (7-39%)

Less common adverse reactions (1% to 10%) include:

Cardiovascular: Hypotension (2-10%), peripheral edema (8%), hypertension (6% to 8%), flushing (5%), edema (<5%)

Central nervous system: Dizziness (10%), anxiety (2% to 5%), agitation (<5%), depression (<5%), hypoesthesia (<5%), insomnia (<5%), malaise (<5%), nervousness (<5%), neuritis (<5%), somnolence (<5%), vertigo (<5%), migraine (RA: 2%)

Dermatologic: Urticaria (2% to 8%)

Endocrine & metabolic: Hyperglycemia (9%), hypoglycemia (<5%), hypercholesterolemia (2%)

Gastrointestinal: Diarrhea (10%), vomiting (10%), dyspepsia (3%), anorexia (<5%), weight loss (<5%)

Hematologic: Anemia (3-8%)

Local: Pain at the injection site (<5%)

Neuromuscular & skeletal: Back pain (10%), myalgia (10%), arthralgia (6% to 10%), paresthesia (2%), arthritis (<5%), hyperkinesia (<5%), hypertonia (<5%), neuropathy (<5%)

Ocular: Conjunctivitis (<5%), lacrimation disorder (<5%)

Respiratory: Throat irritation (2% to 9%), bronchospasm (8%), dyspnea (7%), upper respiratory tract infection (RA: 7%), sinusitis (6%)

Miscellaneous: LDH increased (7%)

Rare events associated with rituximab include severe mucocutaneous reactions, progressive multifocal leukoencephalopathy due to LC virus reactivation, hepatitis B reactivations, and other infections.78

**5.2 ALTERNATIVE TREATMENTS**

The alternative treatments for the subjects participating in this investigation are to continue their medical care under the direction of their attending physician.

**5.3 POTENTIAL BENEFITS**

Participation in the proposed research will not provide a direct benefit to participants in this research. Information obtained from the proposed research will provide information about the relationship between IPF treatment and patient outcome. Potential benefits from the participation in these protocols include enhanced survival, improved respiratory symptoms, and decreased exacerbation severity. Identification of the mechanism(s) mediating these outcomes will facilitate risk-stratification for these adverse outcomes, and development of targeted treatment strategies for the future.

The alternative treatments for the subjects participating in this investigation are to continue their IPF care under the direction of their primary physician.

Based on the preceding assessment of risks and potential benefits, the risks to subjects are reasonable in relation to anticipated benefits. The research presents a balance of risks and expected direct benefits similar to that available in the clinical setting.

Importance of the Knowledge to be Gained

The preliminary data in this application outline a hypothesis for the progressive clinical deterioration in patients with advanced IPF and disease exacerbations. The protocol specifically seeks to address that hypothesis. If the study intervention is found to be both safe and effective in the study population, the treatment of IPF would be altered significantly, and ultimately could lead to a change in the disease natural history. Completion of these protocols will begin to address important questions related to disease.

**5.4 DATA SAFETY MONITORING PLAN**

**5.4.1 Data Safety Monitoring Board**

The local Data Safety Monitoring Board (DSMB) chaired by Dr. Donahoe, the PI of this clinical trial, is comprised of members including senior experts in pulmonary medicine, clinical research, and clinical trial design, biostatistics, and research ethics. The DSMB will conduct interim monitoring of accumulating data from research activities to assure the continue safety of human subjects, relevance and appropriateness of the study, and the integrity of research data.

**5.4.2 Data Safety Monitoring Plan**

Assuring patient safety is an essential component of this protocol. The study Principal Investigator has primary responsibility for the oversight of the data and safety monitoring. The study investigators will evaluate all adverse events. All subjects who have AEs, whether considered associated with the use of the study medication or not, must be monitored to determine the outcome. The clinical course of the AE will be followed up according to accepted standards of medical practice, even after the end of the period of observation, until a satisfactory explanation is found or the Principal Investigator considers it medically justifiable to terminate follow-up.

The study coordinators must view patient records for possible adverse events until 72 hours after the last dose of study drug. All untoward medical occurrences observed in subjects receiving the study drug will be recorded on the participants’ adverse event case report forms (CRF) by the study coordinator under the supervision of the principal investigator. The CRFs will then be reviewed for completeness and internal consistency. Subsequently, the CRFs will be recorded on an electronic password-guarded study database. In addition to internal safeguards built into a computerized system, external safeguards will be put in place to ensure that access to the computerized system and to the data is restricted to authorized personnel. Training conducted by qualified individuals on a continuing basis will be provided to individuals in the specific operations with regard to computerized systems that they are to perform during the course of the study.

The PI, will work with the reporting investigators to prepare a detailed written summary of serious, unexpected, and treatment related adverse events, and will compare, and contrast the event with prior events. The detailed written summary will be provided to the local DSMB and the IRB.

In addition, the DSMB Report addressed the following information will be submitted to the IRB at the time of continuing review annually or more often as required:

- A list of the research personnel who participated in the data and safety monitoring.
- The frequency of monitoring that took place during the renewal intervals and/or the dates that data and safety monitoring was conducted.
- A summary of cumulative data related to unanticipated problems (including adverse events) including a determination of causality and whether the risk to benefit assessment has changed.
- If appropriate, a summary of pertinent scientific literature reports, therapeutic developments, or results of related studies that may have an impact on the safety of study participants or the ethics of the research study.
- A summary of the outcome of reviews conducted to ensure subject privacy and research data confidentiality.
- Final conclusions regarding changes to the anticipated benefit-to-risk assessment of the study participation and final recommendations related to continuing, changing, or terminating the study.

Stopping Rule:

For safety reasons, we propose to discontinue this combined treatment if first 2 of the 10 subjects enrolled in the study experience any unexpected fatal or life-threatening events that can be attributed to the study drug, the study will be halted, until data review by investigators and the Data Safety Monitoring Board has rendered a final recommendation about study continuation.

**5.4.3 Parameters to be Monitored**

The following progress will be monitored throughout the course of the research to ensure the safety of subjects as well as the integrity and confidentiality of their data.

- An evaluation of the progress of the research study, including subject recruitment and retention, and an assessment of the timeliness and quality of the data.
- A review of collected data (including adverse events, unanticipated problems, and subject withdrawals) to determine whether there is a change to the anticipated benefit-to-risk assessment of study participation and whether the study should continue as originally designed, should be changed, or should be terminated.
- An assessment of external factors or relevant information (eg. Pertinent scientific literature reports or therapeutic development, results of related studies) that may have an impact on the safety and study participants or the ethics of the research study.
- A review of study procedures designed to protect the privacy of the research subjects and the confidentiality of their research data.

The National Cancer Institute Common Toxicity Criteria Scale (Appendix 3) will be used to define grades (severity) of adverse events and toxicities. Previous experience with rituximab has demonstrated common side effects that may occur during treatment. An adverse event is any untoward medical occurrence in a participant who received study drug, regardless of its relationship to the study drug. Toxicity is an adverse event with a direct relationship to the study drug. All toxicities are adverse events, but not all adverse events are toxicities. This is a determination made by the study investigator. The study investigators will classify adverse events as “definitely or most likely,” “possibly,” or “very unlikely” due to the study drug. Toxicity will be defined as an adverse event that is definitely, most likely, or possibly caused by the study drug.

The severity of adverse changes in physical signs or symptoms will be classified as follows:

- Grade 1 (Mild): asymptomatic or mild symptoms; clinical or diagnostic observation only; intervention not indicated.
- Grade 2 (Moderate): minimal, local or noninvasive intervention indicated; limiting age-appreciate instrumental ADL (Activities of Daily Living).
- Grade 3 (Severe): medically significant but not immediately life-threatening; hospitalization or prolongation of hospitalization indicated; disabling; limiting self care ADL.
- Grade 4 (Life-threatening): consequences; urgent intervention indicated.
- Grade 5 (Death): event is a direct cause of death.

**5.4.4 Frequency of Monitoring**

The principal investigator will review subject safety data as it generated. The principal investigator, co-principle investigators, and the research staff will meet on a two week interval to re-evaluate study goals, subject recruitment, data coding and retention, documentation and identification of adverse events, complaints and confidentiality of subjects. There will be an evaluation of the progress of the research study, including assessments of data quality, time lines, participant recruitment, accrual, and retention. The principal investigator will also review the outcome and adverse event data to determine whether there is any change to the anticipated benefit-to-risk ratio of study participation and whether the study should continue as originally designed or should it be re-evaluated and changed.

The DSMB will also be expected to meet as needed, but not less than, every six months to provide an overall summary status report to the regulatory agencies.

**5.4.5 Reportable Adverse Events**

For this study, a serious adverse event is any untoward clinical event that is thought by the investigator to be study-related, that is also:

1. Fatal or immediately life threatening

2. Permanently disabling, or severely incapacitating.

3. Requires, or prolongs inpatient hospitalization.

4. Important medical events that may not result in death, be life threatening, or require hospitalization may be considered serious adverse events when, based upon appropriate medical judgment, they may jeopardize the patient, or subject, and may require medical, or surgical intervention to prevent one of the serious outcomes listed above.

If clinically important and unexpected adverse experiences, or clinically important study-related adverse experiences occur, they will be recorded on the adverse event case report form.

**5.4.6 Adverse Events Reporting Timeline**

Life-threatening or fatal unexpected adverse events associated with the use of the study drug or procedures must be reported to the DSMB and the IRB within 24 hours of discovery of the incident with subsequent follow-up submission of a detailed written report.

Serious (but not fatal or life-threatening) and unexpected adverse events associated with the use of the study drug or procedures must be reported to the DSMB and the IRB within 5 working days with subsequent follow-up submission of a detailed written report.

A summary report of the DSMB’s findings will be prepared and submitted to the regulatory agencies.

**5.5 RISKS MANAGEMENT PROCEDURES**

**5.5.1 Protection Against Risks**

General Risks of Study Protocol and Procedures

All research interventions/activities will be conducted in private patient care areas. The collection of sensitive information about subjects is limited to the amount necessary to achieve the aims of the research, so that no unneeded sensitive information is being collected.

All demographic and clinical information about the subject will be stored on an electronic password-guarded study database under the supervision of the PIs for this protocol. The data will be stripped of individual identifiers and stored anonymously with a subject number. Information linking subject identifiers with the coded subject number will be stored under password protection on computers in locked areas, with access only to the database manager. Maintaining records in locked files in locked offices will protect confidentiality of subjects. All staff will sign confidentiality statements. Access to the database will be limited to the data manager and staff under the supervision of the PIs.

Specimens will be stripped of subject identifiers and stored according to a similar coding protocol as described above. These specimens will be stored safely in the custody of the Principal Investigator responsible for the individual assays. These Investigators will limit future access to any remaining sample to only those investigators with prior IRB approval for their studies.

All staff involved in this study are properly credentialed and instructed in the areas of testing, confidentiality, and safety.

The PIs will retain the data for the entire period of this study. The investigators may continue to use and disclose subjects de-identified information for the purpose of this study for a minimum of six years after final reporting or publication of the study. If the subject and/or legal representative decide to withdraw or be withdrawn from study participation, they may request that the study data and samples be destroyed.

**5.5.2 Protection Against Potential Risks of Experimental Intervention**

Despite the documented safety profile of rituximab and plasmapheresis in other human disorders of abnormal immune regulation, the study has been designed with a focus on protecting patients against risk including:

Selection of a target patient population with a very high risk of morbidity and mortality due to the absence of a defined treatment for the disorder

Involvement of trained staff in central venous access placement for the provision of plasmapheresis and utilization of ultrasound guidance for placement of all catheters

Involvement of trained hospital staff for the provision of plasmapheresis in patients with advanced medical illness

Involvement by trained staff / investigators with experience in the administration of Rituximab

Prior human experience with the study medication in similar conditions with an autoimmune hypothesis including rheumatoid arthritis and myositis

Exclusion of all patients with conditions which might simulate IPF exacerbations such as congestive heart failure, pneumonia, and pulmonary thromboembolism

Continuous monitoring by an independent Data and Safety Monitoring Board

Rituximab:

The patient population will receive pre-treatment with acetaminophen (650mg), diphenhydramine (50mg), and methylpredisolone (100mg IV) prior to drug administration. Based upon the experience of the study investigators to date, this regimen will significantly lessen the risk of general reactions to the medication including fever (5% to 53%), chills (3% to 33%), headache (19%), and pain (12%).

Plasmapheresis: The subject will undergo routine monitoring of coagulation parameters (INR) with correction of acquired defects during the procedure consistent with existing medical practice.

Required Education in the Protection of Human Research Participants

All principal and co-investigators listed on University of Pittsburgh Institutional Review Board approved protocol are required to participate in a course entitled The Education and Certification Program in Research & Practice Fundamentals (RPF). This web based tutorial is a requirement of the IRB for protocol submission.

**SECTION 6 – COSTS AND PAYMENTS**

**6.1 COSTS**

Research testing consists of serial T-cell and B-cell subset monitoring, and will be supported by an ongoing research grant focused on longitudinal study of IPF patients (SCCOR 1 P50 HL084932-01). All medications, routine lab tests, and any procedures described will be considered routine medical care and will be billed to the subjects’ health insurance company. Subjects will be responsible for paying any deductibles, co-payments or co-insurance that are a normal part of their health insurance plan. Subjects who do not have health insurance will be responsible for these costs.

**6.2 PAYMENTS**

Participation in this protocol is completely voluntary. Subject will not be compensated in any way for their participation in this research study.

**SECTION 7 – QUALIFICATIONS AND SOURCES OF SUPPORT**

**7.1 QUALIFICATIONS OF THE INVESTIGATORS**

Principal Investigator:

**Michael Donahoe, M.D**., Principal Investigator will provide daily leadership and supervision to all aspects of the clinical trial execution. Dr. Donahoe is an Associate Professor of Medicine, Division of Pulmonary, Allergy, and Critical Care Medicine (PACCM), Department of Medicine, University of Pittsburgh School of Medicine. Dr. Donahoe also serve as the Director of Medical Intensive Care Unit, and has extensive experience in the design and execution of numerous NIH-sponsored complex clinical trials, and co-investigator for studies in nutrition support in COPD, aging in critical care medicine, and the care of critically ill patients. In addition, he has been an active participant in industry sponsored clinical research trials in nutrition support in COPD, ARDS, ICU infections and sepsis investigational drug therapy. He also is uniquely familiar with local and national (FDA and industry) regulatory issues related to the use of aerosol cyclosporine.

Co - Principal Investigators:

**Steven Duncan, M.D**., Pulmonary Physician and Immunologist; He will supervise the T-cell and B-cell expression studies in his laboratory. Dr. Duncan is an Associate Professor of Medicine, Division of PACCM, University of Pittsburgh. Dr. Duncan has longstanding interests in the diagnosis and management of pulmonary disorders.  His research aims to understand the role of adaptive immunity in the pathogenesis of COPD, IPF and lung transplant rejection. Dr. Duncan’s expertise is in the role of T-cells in lung disease, as well as induction of antigen specific tolerance.

**Kevin Gibson, M.D**. Pulmonologist, Dr. Gibson is an Associate Professor of Medicine, Division of PACCM, University of Pittsburgh. Dr. Gibson is the medical director of the Dorothy P. and Richard P. Simmons Center for Interstitial Lung Disease. He is in charge of all clinical research in the center and is the PI on several drug studies. His research interests focus on interstitial lung diseases including IPF, autoimmune lung disease, and sarcoidosis and on translational studies to identify unique biomarkers of disease activity in IPF and other interstitial lung diseases, and studies of gene expression profiling in lung and peripheral blood in IPF.

Co-Investigator:

**Nydia Chien, MSN, RN, CCRC**., Nydia is a research nurse and a Certified Clinical Research Coordinator. She will assist the PI with subject identification, enrollment, follow-up, and data collection.

**7.2 SOURCE OF SUPPORT**

National Heart, Lung, and Blood Institute;

Division of Pulmonary, Allergy, and Critical Care Medicine, University of Pittsburgh.

**SECTION 8 – REFERENCES**

1. American Thoracic Society (2000). Idiopathic pulmonary fibrosis: diagnosis and treatment. International consensus statement. American Thoracic Society (ATS), and the European Respiratory Society (ERS). Am J Respir Crit Care Med 161:646-664. PMID: 10673212

2. [Davies HR](http://www.ncbi.nlm.nih.gov/pubmed?term="Davies HR"%5BAuthor%5D), [Richeldi L](http://www.ncbi.nlm.nih.gov/pubmed?term="Richeldi L"%5BAuthor%5D), Idiopathic pulmonary fibrosis: current and future treatment options. [Am J Respir Med.](javascript:AL_get(this, 'jour', 'Am J Respir Med.');) 2002; 1:211-224. PMID: 14720059

3. Selman, M, Thannickal VJ, Pardo A, Zisman DA, Martinez FJ, Lynch LP. Idiopathic pulmonary fibrosis: pathogenesis and therapeutic approaches. Drugs, 2004; 64:406-430. PMID: 14969575

4. Collard HR, Moore BB, Flaherty KR, Brown KK, Kaner RJ, King, Jr. TE, Lasky JA, Loyd JE, Noth I, Olman MA, Raghu G, Roman J, Ryu JH, Zisman DA, Hunninghake GW, Colby TV, Egan JJ, Hansell DM, Johkoh T, Kaminski N, Kim DS, Kondoh Y, Lynch DA, Mueller-Quernheim J, Myers JL, Nicholson AG, Selman M, Toews GB, Wells AU, Martinez FJ, with the Idiopathic Pulmonary Fibrosis Clinical Research Network Investigators. Acute exacerbations of idiopathic pulmonary fibrosis. Am J Respir Crit Care Med, 2007; 176. 636–643 PMID: 17585107

5. Magro CM, Waldman WJ, Knight DA, Allen JN, Nadasdy T, [Frambach GE](http://www.ncbi.nlm.nih.gov/pubmed?term="Frambach GE"%5BAuthor%5D&itool=EntrezSystem2.PEntrez.Pubmed.Pubmed_ResultsPanel.Pubmed_RVAbstract), [Ross P](http://www.ncbi.nlm.nih.gov/pubmed?term="Ross P"%5BAuthor%5D&itool=EntrezSystem2.PEntrez.Pubmed.Pubmed_ResultsPanel.Pubmed_RVAbstract), [Marsh CB](http://www.ncbi.nlm.nih.gov/pubmed?term="Marsh CB"%5BAuthor%5D&itool=EntrezSystem2.PEntrez.Pubmed.Pubmed_ResultsPanel.Pubmed_RVAbstract). Idiopathic pulmonary fibrosis related to endothelial injury and antiendothelial cell antibodies. Hum Immunol 2006; 67:284-297 PMID: 16720208

6. Ogushi F, Tani K, Endo T, Tada H, Kawano T, Asano T, Huang L, Ohmoto Y, Muraguchi M, Moriguchi H, Sone S. Autoantibodies to IL-1α in sera from rapidly progressive idiopathic pulmonary fibrosis. J Med Invest 2001; 48:181-9 PMID: 11694958

7. Kurosu K, Takiguchi Y, Okada O, Yumoto N, Sakao S, Tada Y, Kasahara Y, Tanabe N, Tatsumi K, Weiden M, Rom WN, Kuriyama T. Identification of annexin 1 as a novel autoantigen in acute exacerbation of idiopathic pulmonary fibrosis. J Immunol 2008; 181:756–767 PMID: 18566442

8. Feghali-Bostwick CA, Tsai CG, Valentine VG, Kantrow S, Stoner MW, [Pilewski JM](http://www.ncbi.nlm.nih.gov/pubmed?term="Pilewski JM"%5BAuthor%5D&itool=EntrezSystem2.PEntrez.Pubmed.Pubmed_ResultsPanel.Pubmed_RVAbstract), [Gadgil A](http://www.ncbi.nlm.nih.gov/pubmed?term="Gadgil A"%5BAuthor%5D&itool=EntrezSystem2.PEntrez.Pubmed.Pubmed_ResultsPanel.Pubmed_RVAbstract), [George MP](http://www.ncbi.nlm.nih.gov/pubmed?term="George MP"%5BAuthor%5D&itool=EntrezSystem2.PEntrez.Pubmed.Pubmed_ResultsPanel.Pubmed_RVAbstract), [Gibson KF](http://www.ncbi.nlm.nih.gov/pubmed?term="Gibson KF"%5BAuthor%5D&itool=EntrezSystem2.PEntrez.Pubmed.Pubmed_ResultsPanel.Pubmed_RVAbstract), [Choi AM](http://www.ncbi.nlm.nih.gov/pubmed?term="Choi AM"%5BAuthor%5D&itool=EntrezSystem2.PEntrez.Pubmed.Pubmed_ResultsPanel.Pubmed_RVAbstract), [Kaminski N](http://www.ncbi.nlm.nih.gov/pubmed?term="Kaminski N"%5BAuthor%5D&itool=EntrezSystem2.PEntrez.Pubmed.Pubmed_ResultsPanel.Pubmed_RVAbstract), [Zhang Y](http://www.ncbi.nlm.nih.gov/pubmed?term="Zhang Y"%5BAuthor%5D&itool=EntrezSystem2.PEntrez.Pubmed.Pubmed_ResultsPanel.Pubmed_RVAbstract), [Duncan SR](http://www.ncbi.nlm.nih.gov/pubmed?term="Duncan SR"%5BAuthor%5D&itool=EntrezSystem2.PEntrez.Pubmed.Pubmed_ResultsPanel.Pubmed_RVAbstract). Cellular and humoral autoreactivity in idiopathic pulmonary fibrosis. J Immunol 2007; 179:2592-2599 PMID: 17675522

9. Gilani SR, Vuga LJ, Lindell KO, Gibson KF, Xue J, Lindsay EK, Kaminski N, Valentine VG, George MP, Steele C, Duncan SR. CD28 down-regulation on circulating CD4 T-cells is associated with poor prognoses of patients with idiopathic pulmonary fibrosis. Plos One 2010; 5:e8959 PMID: 20126467

10. Selva-Ocallaghan A, Labrador-Horrillo M, Munoz-Gall X, Martinez-Gomez X, Majo-Masferrer J, Solans-Laque R, [Simeon-Aznar CP](http://www.ncbi.nlm.nih.gov/pubmed?term="Simeon-Aznar CP"%5BAuthor%5D), [Morell-Brotard F](http://www.ncbi.nlm.nih.gov/pubmed?term="Morell-Brotard F"%5BAuthor%5D), [Vilardell-Tarrés M](http://www.ncbi.nlm.nih.gov/pubmed?term="Vilardell-Tarrés M"%5BAuthor%5D). Polymyositis/dermatomyositis-associated lung disease: analysis of a series of 81 patients. Lupus 2005;14:534–42. PMID: 16130510

11. [Sem M](http://www.ncbi.nlm.nih.gov/pubmed?term="Sem M"%5BAuthor%5D), [Molberg O](http://www.ncbi.nlm.nih.gov/pubmed?term="Molberg O"%5BAuthor%5D), [Lund MB](http://www.ncbi.nlm.nih.gov/pubmed?term="Lund MB"%5BAuthor%5D), [Gran JT](http://www.ncbi.nlm.nih.gov/pubmed?term="Gran JT"%5BAuthor%5D). Rituximab treatment of the anti-synthetase syndrome: a retrospective case series. [Rheumatology (Oxford).](javascript:AL_get(this, 'jour', 'Rheumatology (Oxford).');) 2009; 48:968-971. PMID: 19531628

12. Martinu T, Howell DN, Palmer SM. Acute cellular rejection and humoral sensitization in lung transplant recipients. Semin Resp Crit Care Med. 2010; 31:179-188 PMID: 20354931

13. [Fudala R](http://www.ncbi.nlm.nih.gov/pubmed?term="Fudala R"%5BAuthor%5D), [Krupa A](http://www.ncbi.nlm.nih.gov/pubmed?term="Krupa A"%5BAuthor%5D), [Stankowska D](http://www.ncbi.nlm.nih.gov/pubmed?term="Stankowska D"%5BAuthor%5D), [Allen TC](http://www.ncbi.nlm.nih.gov/pubmed?term="Allen TC"%5BAuthor%5D), [Kurdowska AK](http://www.ncbi.nlm.nih.gov/pubmed?term="Kurdowska AK"%5BAuthor%5D). Does activation of the FcgammaRIIa play a role in the pathogenesis of the acute lung injury/acute respiratory distress syndrome? [Clin Sci (Lond).](javascript:AL_get(this, 'jour', 'Clin Sci (Lond).');) 2010;118(:519-26. PMID: 20088831

14, [Krupa A](http://www.ncbi.nlm.nih.gov/pubmed?term="Krupa A"%5BAuthor%5D), [Kato H](http://www.ncbi.nlm.nih.gov/pubmed?term="Kato H"%5BAuthor%5D), [Matthay MA](http://www.ncbi.nlm.nih.gov/pubmed?term="Matthay MA"%5BAuthor%5D), [Kurdowska AK](http://www.ncbi.nlm.nih.gov/pubmed?term="Kurdowska AK"%5BAuthor%5D) Proinflammatory activity of anti-IL-8 autoantibody:IL-8 complexes in alveolar edema fluid from patients with acute lung injury [Am J Physiol Lung Cell Mol Physiol.](javascript:AL_get(this, 'jour', 'Am J Physiol Lung Cell Mol Physiol.');) 2004 Jun;286(6):L1105-13. PMID: 14729508.

15. Erickson SB, Kurtz SB, Donadio JV, Holley KE, Wilson CB, Pineda AA. Use of combined plasmapharesis and immunosuppression in the treatment of Goodpasture’s syndrome. Mayo Clin Proc 1979; 54:714-720 PMID: 491763

16. Monaco C, Andreakos E, Kiriakidis S, Feldman M, Paleolog C. T-cell-mediated signaling in immune, inflammatory and angiogenic processes: the cascade of events leading to inflammatory diseases. Curr Drug Targets Inflamm Allergy 2004;3:35-42. PMID: 15032640

17. Parker DC. T-cell dependent B-cell activation. Annu Rev Immunol 1993; 11:331-340 PMID: 8476565

18. Rudolph MG, Stanfield RL, Wilson IA. How TCRs bind MHCs, peptides, and co-receptors. Annu Rev Immunol 2006, 24:419-466 PMID: 16551255

19. Ermann J, Fathman CG. Autoimmune diseases: genes, bugs, and failed regulation. Nat Immunol 2001; 2:759-761 PMID: 11526377

20. Marrack P, Kappler J, Kotzin BL. Autoimmune disease: why and where it occurs. Nat Immunol 2001; 7:899-905 PMID: 11479621

21. Lipsky PE. Systemic lupus erythematosus: an autoimmune disease of B cell hyperactivity. Nat Immunol 2001; 2:764-766 PMID: 11526379

22. Hoshida S, Nishion M, Tanouchi J, Kishimoto T, Yamad Y. Acute Chlamydia pneumonia infection with heat shock protein 60-related response in patients with acute coronary syndromes. Atherosclerosis 2005; 183:109-112. PMID: 16216593

23. Vanderlugt CL, Miller SD. Epitope spreading in immune mediated diseases: implications for immunotherapy. Nat Rev Immunol 2002, 2:85-94 PMID: 11910899

24. Oldstone MB. Molecular mimicry, microbial infection and autoimmune disease: evolution of the concept. Curr Top Microbiol Immunol 2005, 296:1-17 PMID: 16329189

25. Hall FC, Bowness P. HLA and disease: From molecular function to disease association? In: Browning MJ, McMichael AJ (eds) HLA and MHC: Genes, molecules, and function, BIOS Scientific Publishers Ltd, Oxford, UK. 1996, pp 353-381.

26. Warrens A, Lechler R. HLA in health and disease.1999, Academic Press, San Diego, CA.

27. Takeuchi F, Nakano K, Nabeta H, Hong GH, Kawasugi K, Mori M, Okudaira H, Kuwata S, Tanimoto K. Genetic contribution of the tumor necrosis factor (TNF) B + 252*2/2 genotype, but not the TNFa,b microsatellite alleles, to system lupus erythematosis in Japanese patients. Int J Immunognet 2005; 32:173-178 PMID: 15932622

28. de Bakker PI, McVean G, Sabeti PC, Miretti MM, Green T, Marchini J, Ke X, Monsuur AJ, Whittaker P, Delgado M, Morrison J, Richardson A, Walsh EC, Gao X, Galver L, Hart J, Hafler DA, Pericak-Vance M, Todd JA, Daly MJ, Trowsdale J, Wijmenga C, Vyse TJ, Beck S, Murray SS, Carrington M, Gregory S, Deloukas P, Rioux JD. A high-resolution HLA and SNP haplotype map for disease association studies in the extended human MHC. Nature Genetics, 2006, 38:1166-1172 PMID: 16998491

29. Schmidt H, Williamson D, Ashley-Koch A. HLA-DR15 haplotype and multiple sclerosis: a huge review. Am J Epidemiol 2007; 165:1097-1109 PMID: 17329717

30. Voorter CEM, Drent M, van den Berg-Loonen EM. Severe pulmonary sarcoidosis is strongly associated with the haplotype HLA-DQB1*0602-DRB1*1501. Hum Immunol 2005; 66:826-835 PMID: 16112030

31. Xue J, Gochuico BR, Kahloon RA, Bhargava A, Alawas AS, Feghali-Bostwick CA, Nathan SD, Rosen GD, Rosas IO, Csizmadia E, Dacic S, Ocak I, Fuhrman CR, Cuenco KT, Smith MA, Jacobs SS, Zeevi A, Morel PA, Pilewski JM, Sciurba FC, Zhang Y, Duncan SR. The HLA allele DRB1*15 is over-represented in patients with idiopathic pulmonary fibrosis. Revision submitted (PlosOne).

32, National Bone Marrow Donor Program. Haplotype Frequencies. Accessed June 10, 2010 at https://bioinformatics.nmdp.org.

33. Evans C. HLA antigens in diffuse fibrosing alveolitis. Thorax 1976; 31:483-5 doi:10.1136/thx.31.4.480

34. Strimlan CV, Taswell HF, DeRemee RA, Kueppers F. HLA antigens and fibrosing alveolitis. Am Rev Resp Dis 1977; 1120-1 PMID: 931187

35. Fulmer JD, Sposovska MS, von Gal ER, Crystal RG, Mittal KK. Distribution of HLA antigens in idiopathic pulmonary fibrosis. Am Rev Resp Dis 1978; 118:141-47 PMID: 677553

36. Turton CWG, Morris LM, Lawler SD, Turner-Warwick M. HLA in cryptogenic fibrosing alveolitis. Lancet 1978; 1(8062):507-8 PMID: 76058

37. Varpela E., Tiilkainen A, Varpela M, Tukiainen P. High prevalences of HLA-B15 and HLA-Dw6 in patients with cryptogenic fibrosing alveolitis. Tissue Antigens 1979; 14:68-71 PMID: 91222

38. Libby DM, Gibofsky A, Fotino M, Waters SJ, Smith JP. Immunogenetic and clinical findings in idiopathic pulmonary fibrosis. Am Rev Resp Dis 1983; 127:618-22 PMID: 6405666

39. Falfan-Valenci R, Camarena A, Juarez A, Becerril C, Montano M, Cisneros J, Mendoza F, Granados J, Pardo A, Selman M. Major histocompatibility complex and alveolar epithelial apoptosis in idiopathic pulmonary fibrosis. Hum Genet 2005; 118:235-244 PMID: 16133177

40. Rosas IO, Ren P, Avila NA, Chow CK, Franks TJ, Travis WD, McCoy JP Jr, May RM, Wu HP, Nguyen DM, Arcos-Burgos M, MacDonald SD, Gochuico BR. Early interstitial lung disease in familial pulmonary fibrosis. Am J Resp Crit Care Med 2007; 176:698-705. PMID: 17641157

41. Kotslanidis I, Nakou E, Bouchliou I, Tzouvelekis A, Spanoudakis E, [Steiropoulos P](http://www.ncbi.nlm.nih.gov/pubmed?term="Steiropoulos P"%5BAuthor%5D&itool=EntrezSystem2.PEntrez.Pubmed.Pubmed_ResultsPanel.Pubmed_RVAbstract), [Sotiriou I](http://www.ncbi.nlm.nih.gov/pubmed?term="Sotiriou I"%5BAuthor%5D&itool=EntrezSystem2.PEntrez.Pubmed.Pubmed_ResultsPanel.Pubmed_RVAbstract), [Aidinis V](http://www.ncbi.nlm.nih.gov/pubmed?term="Aidinis V"%5BAuthor%5D&itool=EntrezSystem2.PEntrez.Pubmed.Pubmed_ResultsPanel.Pubmed_RVAbstract), [Margaritis D](http://www.ncbi.nlm.nih.gov/pubmed?term="Margaritis D"%5BAuthor%5D&itool=EntrezSystem2.PEntrez.Pubmed.Pubmed_ResultsPanel.Pubmed_RVAbstract), [Tsatalas C](http://www.ncbi.nlm.nih.gov/pubmed?term="Tsatalas C"%5BAuthor%5D&itool=EntrezSystem2.PEntrez.Pubmed.Pubmed_ResultsPanel.Pubmed_RVAbstract), [Bouros D](http://www.ncbi.nlm.nih.gov/pubmed?term="Bouros D"%5BAuthor%5D&itool=EntrezSystem2.PEntrez.Pubmed.Pubmed_ResultsPanel.Pubmed_RVAbstract). Global impairment of CD4^+^CD25^+^FoxP3^+^ regulatory T cells in idiopathic pulmonary fibrosis. Am J Resp Crit Care Med 2009; 179:1121-1130 PMID: 19342412

42 Shimizudani A, Murata H, Keino H, Kojo S, Nakamura H, [Morishima Y](http://www.ncbi.nlm.nih.gov/pubmed?term="Morishima Y"%5BAuthor%5D&itool=EntrezSystem2.PEntrez.Pubmed.Pubmed_ResultsPanel.Pubmed_RVAbstract), [Sakamoto T](http://www.ncbi.nlm.nih.gov/pubmed?term="Sakamoto T"%5BAuthor%5D&itool=EntrezSystem2.PEntrez.Pubmed.Pubmed_ResultsPanel.Pubmed_RVAbstract), [Ohtsuka M](http://www.ncbi.nlm.nih.gov/pubmed?term="Ohtsuka M"%5BAuthor%5D&itool=EntrezSystem2.PEntrez.Pubmed.Pubmed_ResultsPanel.Pubmed_RVAbstract), [Sekisawa K](http://www.ncbi.nlm.nih.gov/pubmed?term="Sekisawa K"%5BAuthor%5D&itool=EntrezSystem2.PEntrez.Pubmed.Pubmed_ResultsPanel.Pubmed_RVAbstract), [Sumida M](http://www.ncbi.nlm.nih.gov/pubmed?term="Sumida M"%5BAuthor%5D&itool=EntrezSystem2.PEntrez.Pubmed.Pubmed_ResultsPanel.Pubmed_RVAbstract), [Sumida T](http://www.ncbi.nlm.nih.gov/pubmed?term="Sumida T"%5BAuthor%5D&itool=EntrezSystem2.PEntrez.Pubmed.Pubmed_ResultsPanel.Pubmed_RVAbstract), [Matsuoka T](http://www.ncbi.nlm.nih.gov/pubmed?term="Matsuoka T"%5BAuthor%5D&itool=EntrezSystem2.PEntrez.Pubmed.Pubmed_ResultsPanel.Pubmed_RVAbstract). Conserved CDR 3 region of T cell receptor BV gene in lymphocytes from bronchoalveolar lavage fluid of patients with idiopathic pulmonary fibrosis. Clin Exp Immunol 2002; 129:140-149. PMID: 12100034

43. Maini MK, Casorati G, Dellabona P, Waxk A, Beverely PCL. T-cell clonality in immune responses. Immunol. Today, 1999, 20:161-166. PMID: 10354551

44. Vallejo AN, Weyand CM, Goronzy JJ. T-cell senescence: a culprit of immune abnormalities in chronic inflammation and persistent infection. Trends Mol Med 2004;10:119-124. PMID: 15102354

45. Studer SM, George MP, Zhu X, Song Y, Valentine VG, Stoner MW, Sethi JM, Steele C, Duncan SR. CD28 downregulation on CD4 T-Cells is a marker for graft dysfunction in lung transplant recipients. Am J Resp Critical Care Med. 2008; 178:765-773. PMID: 18617642

46. Liuzzo G., J. J. Goronzy, H. Yang, S. L. Kopecky, D. R. Holmes, R. L. Frye, and C. M. Weyand. Monoclonal T-cell proliferation and plaque instability in acute coronary syndromes. [Circulation.](javascript:AL_get(this, 'jour', 'Circulation.');) 2000 Jun 27;101(25):2883-8. PMID: 10869258

47. Martens PB, Goronzy JJ, Schaid D, Weyand CM. Expansion of unusual CD4+ T cells in severe rheumatoid arthritis. Arth and Rhem. 1997. 40:1106-14 PMID: 9182921

48. Goronzy J J, Matteson EL, Fulbright JW, Warrington KJ, Chang-Miller A, Hunder G, Mason TG, Nelson AM, Valente RM, Crowson CS, Erlich HA, Reynolds RL, Swee RG, O'Fallon WM, Weyand CM. Prognostic markers of radiographic progression in early rheumatoid arthritis. Arthritis Rheum, 2004; 50:43-54 PMID: 14730598

49. Manuscript in preparation

50. Campbell DA, Poulter LW, Janossy G, du Bois RM. Immunohistological analysis of lung tissue from patients with cryptogenic fibrosing alveolitis suggesting local expression of immune hypersensitivity. Thorax 1985;40:405-11. PMID: 3875162

51. Marchal-Somme J, Uzunhan Y, Marchand-Adam S, Valeyre D, Soumelis V, [Crestani B](http://www.ncbi.nlm.nih.gov/pubmed?term="Crestani B"%5BAuthor%5D&itool=EntrezSystem2.PEntrez.Pubmed.Pubmed_ResultsPanel.Pubmed_RVAbstract), [Soler P](http://www.ncbi.nlm.nih.gov/pubmed?term="Soler P"%5BAuthor%5D&itool=EntrezSystem2.PEntrez.Pubmed.Pubmed_ResultsPanel.Pubmed_RVAbstract). Cutting edge: non-proliferating mature immune cells form a novel type of organizing lymphoid structure in idiopathic pulmonary fibrosis. J Immunol 2006; 176:5735-5739 PMID: 16670278

52. Parra ER, Kairalla RA, Ribeiro de Carvalho CR, Eher E, Capelozzi VL. Inflammatory cell phenotyping of the pulmonary interstitium in idiopathic interstitial pneumonia. Respiration 2007, 74:159-69 PMID: 17108669

53. Zuo F, Kaminski N, Eugui E, Allard J, Hakhini Z, Ben-Dor A, Lollini L, Morris D, Kim Y, DeLustro B, Sheppard D, Pardo A, Selman M, Heller RA. Gene expression analysis reveals matrilysin as a key regulator of pulmonary fibrosis in mice and humans. Proc Natl Acad Sci., USA, 2002; 99:6292-97 PMID: 11983918

54. Dall Aglio PP, Pesci A, Bertorelli G, Brianti E, Scarpa S. Study of immune complexes in broncholaveolar lavage fluids. Respiration 1988; 54:36-41 PMID: 3231904

55. Dobashi N, Fujita J, Murota M, Ohtsuki Y, Yamadori I, Yoshinouchi T, Ueda R, Bandoh S, Kamei T, Nishioka M, Ishida T, Takahara J. Elevation of anti-cytokeratin 18 antibody and circulating cytokeratin 18: anti-cytokeratin 18 antibody immune complexes in sera of patients with idiopathic pulmonary fibrosis. Lung 2000;178:171-9 PMID: 10871435

56. Grigolo B, Mazzetti I, Borzi RM, Hickson ID, Fabbri M, Fasano L, Meliconi R, Facchini A. Mapping of topoisomerase II alpha epitopes recognized by autoantibodies in idiopathic pulmonary fibrosis. Clin Exp Immunol 1998;114: 339-46. PMID: 9844041

57. Yang Y, Fujita J, Bandho S, Ohtsuki Y, Yamadori I, Yoshinouchi T, Ishida T. Detection of antivimentin antibody in sera of patients with idiopathic pulmonary fibrosis and non-specific interstitial pneumonia. Clin Exp Immunol 2002;128:169-74. PMID: 11982605

58. Takahashi T, Wada I, Ohtsuka Y, Munakata M, Homm Y, Kuroki Y. Autoantibody to alanyl-tRNA synthetase in patients with idiopathic pulmonary fibrosis. Respirology 2007; 12:642-653. PMID: 17875050

59. Wallace WAH, Howie SM. Upregulation of tenascin and TGF-β production in a type II alveolar epithelial cell line by antibody against a pulmonary auto-antigen. J Pathol 2001;195:251-6 PMID: 11592106

60. Jang JY, Jeong JG, Jun HR, Lee SC, Kim JS, Kim YS, Kwon MH. [A nucleic acid-hydrolyzing antibody penetrates into cells via caveolae-mediated endocytosis, localizes in the cytosol and exhibits cytotoxicity.](http://www.ncbi.nlm.nih.gov/pubmed/19373434) Cell Mol Life Sci. 2009 Jun;66(11-12):1985-97. PMID: 19373434

61. Gonzalez-Grwonow M, Cuchacovich M, Llanos C, Urzua C, Gawdi G, Pizzo SV. Prostate cancer cell proliferation in vitro is modulated by antibodies against glucose-regulated protein 78 isolated from patient serum. Cancer Res 2006; 66:11424-11431 PMID: 17145889

62. [Lu MC](http://www.ncbi.nlm.nih.gov/pubmed?term="Lu MC"%5BAuthor%5D), [Lai NS](http://www.ncbi.nlm.nih.gov/pubmed?term="Lai NS"%5BAuthor%5D), [Yu HC](http://www.ncbi.nlm.nih.gov/pubmed?term="Yu HC"%5BAuthor%5D), [Huang HB](http://www.ncbi.nlm.nih.gov/pubmed?term="Huang HB"%5BAuthor%5D), [Hsieh SC](http://www.ncbi.nlm.nih.gov/pubmed?term="Hsieh SC"%5BAuthor%5D), [Yu CL](http://www.ncbi.nlm.nih.gov/pubmed?term="Yu CL"%5BAuthor%5D). Anti-citrullinated protein antibodies bind surface-expressed citrullinated Grp78 on monocyte/macrophages and stimulate tumor necrosis factor alpha production. [Arthritis Rheum.](javascript:AL_get(this, 'jour', 'Arthritis Rheum.');) 2010 62:1213-23. PMID: 16265688

63. Feghali-Bostwick CA, Gadgil AS, Otterbein LE, Pilewski JM, Stoner MW, Csizmadia E, Zhang Y, Sciurba FC, Duncan SR. Autoantibodies in patients with chronic obstructive pulmonary disease. Am J Resp Critical Care Med 2008;177:156-163. PMID: 17975205

64. Mayada TN, Tsokos GC, Tsuboi N. Mechanisms of immune complex-mediated neutrophil recruitment and tissue injury. Circulation 2009; 120:2012-2024 PMID: 19917895

65. Prohászka Z. [Chaperones as part of immune networks.](http://www.ncbi.nlm.nih.gov/pubmed/17205683?itool=EntrezSystem2.PEntrez.Pubmed.Pubmed_ResultsPanel.Pubmed_RVDocSum&ordinalpos=19) Adv Exp Med Biol. 2007: 594:159-66 PMID: 17205683

66. Tahiri F, Le Naour F, Huguet S, Lai-Kuen R, Samuel D, Johanet C, Saubamea B, Tricottet V, Duclos-Vallee JC, Ballot E. [Identification of plasma membrane autoantigens in autoimmune hepatitis type 1 using a proteomics tool](http://www.ncbi.nlm.nih.gov/pubmed/18306218?itool=EntrezSystem2.PEntrez.Pubmed.Pubmed_ResultsPanel.Pubmed_RVDocSum&ordinalpos=4) Hepatology. 2008; 47:937-48 PMID: 18306218

67. Zlacka D, Vavrincova P, Hien Nguyen TT, Hromadnikova I. [Frequency of anti-hsp60, -65 and -70 antibodies in sera of patients with juvenile idiopathic arthritis.](http://www.ncbi.nlm.nih.gov/pubmed/16934956?itool=EntrezSystem2.PEntrez.Pubmed.Pubmed_ResultsPanel.Pubmed_RVDocSum&ordinalpos=8) J Autoimmun. 2006;27:81-8. PMID: 16934956

68. [Abulafia-Lapid R](http://www.ncbi.nlm.nih.gov/pubmed?term="Abulafia-Lapid R"%5BAuthor%5D), [Gillis D](http://www.ncbi.nlm.nih.gov/pubmed?term="Gillis D"%5BAuthor%5D), [Yosef O](http://www.ncbi.nlm.nih.gov/pubmed?term="Yosef O"%5BAuthor%5D), [Atlan H](http://www.ncbi.nlm.nih.gov/pubmed?term="Atlan H"%5BAuthor%5D), [Cohen IR](http://www.ncbi.nlm.nih.gov/pubmed?term="Cohen IR"%5BAuthor%5D). T cells and autoantibodies to human HSP70 in Type 1 diabetes in children. J Autoimmun 2003; 20:313–321 PMID: 12791317

69. [Pozsonyi E](http://www.ncbi.nlm.nih.gov/pubmed?term="Pozsonyi E"%5BAuthor%5D), [György B](http://www.ncbi.nlm.nih.gov/pubmed?term="György B"%5BAuthor%5D), [Berki T](http://www.ncbi.nlm.nih.gov/pubmed?term="Berki T"%5BAuthor%5D), [Bánlaki Z](http://www.ncbi.nlm.nih.gov/pubmed?term="Bánlaki Z"%5BAuthor%5D), [Buzás E](http://www.ncbi.nlm.nih.gov/pubmed?term="Buzás E"%5BAuthor%5D), [Rajczy K](http://www.ncbi.nlm.nih.gov/pubmed?term="Rajczy K"%5BAuthor%5D), [Hossó A](http://www.ncbi.nlm.nih.gov/pubmed?term="Hossó A"%5BAuthor%5D), [Prohászka Z](http://www.ncbi.nlm.nih.gov/pubmed?term="Prohászka Z"%5BAuthor%5D), [Szilágyi A](http://www.ncbi.nlm.nih.gov/pubmed?term="Szilágyi A"%5BAuthor%5D), [Cervenak L](http://www.ncbi.nlm.nih.gov/pubmed?term="Cervenak L"%5BAuthor%5D), [Füst G](http://www.ncbi.nlm.nih.gov/pubmed?term="Füst G"%5BAuthor%5D). HLA-association of serum levels of natural antibodies. [Mol Immunol.](javascript:AL_get(this, 'jour', 'Mol Immunol.');) 2009;46 :1416-1423. PMID: 19167759

70. Kelsen SG, Duan X, Rong J, Perez O, Liu C, Merali S. Cigarette smoke induces an unfolded protein response in the human lung. Am J Resp Cell Mol Bio. 2008; 38: 541-550 PMID: 18079489

71. Lee S-H, Goswami S, Grudo A, Song L-Z, Bandi V, Goodnight-White S, Green L, Hacken-Bitar J, Huh J, Bakaeen F, Coxson HO, Cogswell S, Storness-Bliss C, Corry DB, Kheradmand K. Antielastin autoimmunity in tobacco smoking-induced emphysema. Nat Med 2007; 13:567-9 PMID: 17450149

72. [Bottazzo GF](http://www.ncbi.nlm.nih.gov/pubmed?term="Bottazzo GF"%5BAuthor%5D), [Doniach D](http://www.ncbi.nlm.nih.gov/pubmed?term="Doniach D"%5BAuthor%5D). Autoimmune thyroid disease. [Annu Rev Med.](javascript:AL_get(this, 'jour', 'Annu Rev Med.');) 1986;37:353-9.PMID: 2871804

73. Wynn TA. Fibrotic disease and the TH1/TH2 paradigm. Nat Immunol Rev, 2004, 4:583-594 PMID: 15286725

74. [Perosa F](http://www.ncbi.nlm.nih.gov/pubmed?term="Perosa F"%5BAuthor%5D), [Prete M](http://www.ncbi.nlm.nih.gov/pubmed?term="Prete M"%5BAuthor%5D), [Racanelli V](http://www.ncbi.nlm.nih.gov/pubmed?term="Racanelli V"%5BAuthor%5D), [Dammacco F](http://www.ncbi.nlm.nih.gov/pubmed?term="Dammacco F"%5BAuthor%5D). CD20-depleting therapy in autoimmune diseases: from basic research to the clinic. [J Intern Med.](javascript:AL_get(this, 'jour', 'J Intern Med.');) 2010, 267:260-77 PMID: 20201920

75. [Jordan SC](http://www.ncbi.nlm.nih.gov/pubmed?term="Jordan SC"%5BAuthor%5D), [Reinsmoen N](http://www.ncbi.nlm.nih.gov/pubmed?term="Reinsmoen N"%5BAuthor%5D), [Peng A](http://www.ncbi.nlm.nih.gov/pubmed?term="Peng A"%5BAuthor%5D), [Lai CH](http://www.ncbi.nlm.nih.gov/pubmed?term="Lai CH"%5BAuthor%5D), [Cao K](http://www.ncbi.nlm.nih.gov/pubmed?term="Cao K"%5BAuthor%5D), [Villicana R](http://www.ncbi.nlm.nih.gov/pubmed?term="Villicana R"%5BAuthor%5D), [Toyoda M](http://www.ncbi.nlm.nih.gov/pubmed?term="Toyoda M"%5BAuthor%5D), [Kahwaji J](http://www.ncbi.nlm.nih.gov/pubmed?term="Kahwaji J"%5BAuthor%5D), [Vo AA](http://www.ncbi.nlm.nih.gov/pubmed?term="Vo AA"%5BAuthor%5D).

# Advances in diagnosing and managing antibody-mediated rejection. [**Pediatr Nephrol.**](javascript:AL_get(this, 'jour', 'Pediatr Nephrol.');) 2010 25::2035-45

76. [Stone JH](http://www.ncbi.nlm.nih.gov/pubmed?term="Stone JH"%5BAuthor%5D), [Merkel PA](http://www.ncbi.nlm.nih.gov/pubmed?term="Merkel PA"%5BAuthor%5D), [Spiera R](http://www.ncbi.nlm.nih.gov/pubmed?term="Spiera R"%5BAuthor%5D), [Seo P](http://www.ncbi.nlm.nih.gov/pubmed?term="Seo P"%5BAuthor%5D), [Langford CA](http://www.ncbi.nlm.nih.gov/pubmed?term="Langford CA"%5BAuthor%5D), [Hoffman GS](http://www.ncbi.nlm.nih.gov/pubmed?term="Hoffman GS"%5BAuthor%5D), [Kallenberg CG](http://www.ncbi.nlm.nih.gov/pubmed?term="Kallenberg CG"%5BAuthor%5D), [St Clair EW](http://www.ncbi.nlm.nih.gov/pubmed?term="St Clair EW"%5BAuthor%5D), [Turkiewicz A](http://www.ncbi.nlm.nih.gov/pubmed?term="Turkiewicz A"%5BAuthor%5D), [Tchao NK](http://www.ncbi.nlm.nih.gov/pubmed?term="Tchao NK"%5BAuthor%5D), [Webber L](http://www.ncbi.nlm.nih.gov/pubmed?term="Webber L"%5BAuthor%5D), [Ding L](http://www.ncbi.nlm.nih.gov/pubmed?term="Ding L"%5BAuthor%5D), [Sejismundo LP](http://www.ncbi.nlm.nih.gov/pubmed?term="Sejismundo LP"%5BAuthor%5D), [Mieras K](http://www.ncbi.nlm.nih.gov/pubmed?term="Mieras K"%5BAuthor%5D), [Weitzenkamp D](http://www.ncbi.nlm.nih.gov/pubmed?term="Weitzenkamp D"%5BAuthor%5D), [Ikle D](http://www.ncbi.nlm.nih.gov/pubmed?term="Ikle D"%5BAuthor%5D), [Seyfert-Margolis V](http://www.ncbi.nlm.nih.gov/pubmed?term="Seyfert-Margolis V"%5BAuthor%5D), [Mueller M](http://www.ncbi.nlm.nih.gov/pubmed?term="Mueller M"%5BAuthor%5D), [Brunetta P](http://www.ncbi.nlm.nih.gov/pubmed?term="Brunetta P"%5BAuthor%5D), [Allen NB](http://www.ncbi.nlm.nih.gov/pubmed?term="Allen NB"%5BAuthor%5D), [Fervenza FC](http://www.ncbi.nlm.nih.gov/pubmed?term="Fervenza FC"%5BAuthor%5D), [Geetha D](http://www.ncbi.nlm.nih.gov/pubmed?term="Geetha D"%5BAuthor%5D), [Keogh KA](http://www.ncbi.nlm.nih.gov/pubmed?term="Keogh KA"%5BAuthor%5D), [Kissin EY](http://www.ncbi.nlm.nih.gov/pubmed?term="Kissin EY"%5BAuthor%5D), [Monach PA](http://www.ncbi.nlm.nih.gov/pubmed?term="Monach PA"%5BAuthor%5D), [Peikert T](http://www.ncbi.nlm.nih.gov/pubmed?term="Peikert T"%5BAuthor%5D), [Stegeman C](http://www.ncbi.nlm.nih.gov/pubmed?term="Stegeman C"%5BAuthor%5D), [Ytterberg SR](http://www.ncbi.nlm.nih.gov/pubmed?term="Ytterberg SR"%5BAuthor%5D), [Specks U](http://www.ncbi.nlm.nih.gov/pubmed?term="Specks U"%5BAuthor%5D); [RAVE-ITN Research Group](http://www.ncbi.nlm.nih.gov/pubmed?term="RAVE-ITN Research Group"%5BCorporate Author%5D). Rituximab versus cyclophosphamide for ANCA-associated vasculitis. New [Engl J Med.](javascript:AL_get(this, 'jour', 'N Engl J Med.');) 2010; 363:221-32. PMID: 20077121

77. [Koulova L](http://www.ncbi.nlm.nih.gov/pubmed?term="Koulova L"%5BAuthor%5D), [Alexandrescu D](http://www.ncbi.nlm.nih.gov/pubmed?term="Alexandrescu D"%5BAuthor%5D), [Dutcher JP](http://www.ncbi.nlm.nih.gov/pubmed?term="Dutcher JP"%5BAuthor%5D), [O'Boyle KP](http://www.ncbi.nlm.nih.gov/pubmed?term="O'Boyle KP"%5BAuthor%5D), [Eapen S](http://www.ncbi.nlm.nih.gov/pubmed?term="Eapen S"%5BAuthor%5D), [Wiernik PH](http://www.ncbi.nlm.nih.gov/pubmed?term="Wiernik PH"%5BAuthor%5D)**.** Rituximab for the treatment of refractory idiopathic thrombocytopenic purpura (ITP) and thrombotic thrombocytopenic purpura (TTP): report of three cases. [Am J Hematol.](javascript:AL_get(this, 'jour', 'Am J Hematol.');) 2005 Jan;78(1):49-54. PMID: 15609292

78. Rituximab package insert and prescribing information, accessed August 28, 2010: [www.gene.com/gene/products/information/pdf/rituxan-prescribing.pdf](http://www.gene.com/gene/products/information/pdf/rituxan-prescribing.pdf)

79. Bambauer R, Latza R, Lentz MR. Therapeutic Plasma Exchange and Selective Plasma Separation- Fundamental Technologies, Pathology, and Clinical Results. 3^rd^ Ed. Pabst-Science Publishers. Lengerich, Germany, 2009, 428 pp. ISBN 978-3-89967-458-3

80. [McGee DC](http://www.ncbi.nlm.nih.gov/pubmed?term="McGee DC"%5BAuthor%5D), [Gould MK](http://www.ncbi.nlm.nih.gov/pubmed?term="Gould MK"%5BAuthor%5D). Preventing complications of central venous catheterization. N. [Engl J Med.](javascript:AL_get(this, 'jour', 'N Engl J Med.');) 2003 Mar 20;348(12):1123-33. PMID: 12646670

81. [Silverman GJ](http://www.ncbi.nlm.nih.gov/pubmed?term="Silverman GJ"%5BAuthor%5D), [Carson DA](http://www.ncbi.nlm.nih.gov/pubmed?term="Carson DA"%5BAuthor%5D). Roles of B cells in rheumatoid arthritis. [Arthritis Res Ther.](javascript:AL_get(this, 'jour', 'Arthritis Res Ther.');) 2003**;**5 Suppl 4:S1-6. PMID: 15180890

82. [Wilk E](http://www.ncbi.nlm.nih.gov/pubmed?term="Wilk E"%5BAuthor%5D), [Witte T](http://www.ncbi.nlm.nih.gov/pubmed?term="Witte T"%5BAuthor%5D), [Marquardt N](http://www.ncbi.nlm.nih.gov/pubmed?term="Marquardt N"%5BAuthor%5D), [Horvath T](http://www.ncbi.nlm.nih.gov/pubmed?term="Horvath T"%5BAuthor%5D), [Kalippke K](http://www.ncbi.nlm.nih.gov/pubmed?term="Kalippke K"%5BAuthor%5D), [Scholz K](http://www.ncbi.nlm.nih.gov/pubmed?term="Scholz K"%5BAuthor%5D), [Wilke N](http://www.ncbi.nlm.nih.gov/pubmed?term="Wilke N"%5BAuthor%5D), [Schmidt RE](http://www.ncbi.nlm.nih.gov/pubmed?term="Schmidt RE"%5BAuthor%5D), [Jacobs R](http://www.ncbi.nlm.nih.gov/pubmed?term="Jacobs R"%5BAuthor%5D)

Depletion of functionally active CD20+ T cells by rituximab treatment. [Arthritis Rheum.](javascript:AL_get(this, 'jour', 'Arthritis Rheum.');) 2009;60:3563-71. PMID: 19950291

83. [Stasi R](http://www.ncbi.nlm.nih.gov/pubmed?term="Stasi R"%5BAuthor%5D). Rituximab in autoimmune hematologic diseases: not just a matter of B cells. [Semin Hematol.](javascript:AL_get(this, 'jour', 'Semin Hematol.');) 2010;47:170-9. PMID: 20350664

84. [Cambridge G](http://www.ncbi.nlm.nih.gov/pubmed?term="Cambridge G"%5BAuthor%5D), [Leandro MJ](http://www.ncbi.nlm.nih.gov/pubmed?term="Leandro MJ"%5BAuthor%5D), [Edwards JC](http://www.ncbi.nlm.nih.gov/pubmed?term="Edwards JC"%5BAuthor%5D), [Ehrenstein MR](http://www.ncbi.nlm.nih.gov/pubmed?term="Ehrenstein MR"%5BAuthor%5D), [Salden M](http://www.ncbi.nlm.nih.gov/pubmed?term="Salden M"%5BAuthor%5D), [Bodman-Smith M](http://www.ncbi.nlm.nih.gov/pubmed?term="Bodman-Smith M"%5BAuthor%5D), [Webster AD](http://www.ncbi.nlm.nih.gov/pubmed?term="Webster AD"%5BAuthor%5D). Serologic changes following B lymphocyte depletion therapy for rheumatoid arthritis. [Arthritis Rheum.](javascript:AL_get(this, 'jour', 'Arthritis Rheum.');) 2003; 48:2146-54. PMID: 12905467

85. [Ahmed AR](http://www.ncbi.nlm.nih.gov/pubmed?term="Ahmed AR"%5BAuthor%5D), [Spigelman Z](http://www.ncbi.nlm.nih.gov/pubmed?term="Spigelman Z"%5BAuthor%5D), [Cavacini LA](http://www.ncbi.nlm.nih.gov/pubmed?term="Cavacini LA"%5BAuthor%5D), [Posner MR](http://www.ncbi.nlm.nih.gov/pubmed?term="Posner MR"%5BAuthor%5D).Treatment of pemphigus vulgaris with rituximab and intravenous immune globulin. [N Engl J Med.](javascript:AL_get(this, 'jour', 'N Engl J Med.');) 2006;355:1772-9. PMID: 17065638

86 Arzoo K, Sadeghi S, Liebman HA.[Treatment of refractory antibody mediated autoimmune disorders with an anti-CD20 monoclonal antibody (rituximab).](http://www.ncbi.nlm.nih.gov/pubmed/12228164)Ann Rheum Dis. 2002; 61:922-4. PMID: 12228164

87. Kelishadi SS, Azimzadeh AM, Zhang T, Stoddard T, Welty E, Avon C, Higuchi M, Laaris A, Cheng XF, McMahon C, Pierson RN 3rd. [Preemptive CD20+ B cell depletion attenuates cardiac allograft vasculopathy in cyclosporine-treated monkeys.](http://www.ncbi.nlm.nih.gov/pubmed/20335656) J Clin Invest. 2010;120:1275-84. PMID: 20335656

88. Han, MK, Murray S, Fell CD, Flaherty KR, Toews GB, Myers J, Colby WD, Travis EA, Kazerooni EA, Gross BH, Martinez FJ.  Sex differences in physiological progression of idiopathic pulmonary fibrosis. Eur Respir J 2008;31:1183-8
